# Evaluation of single-cell RNAseq labelling algorithms using cancer datasets

**DOI:** 10.1093/bib/bbac561

**Published:** 2022-12-30

**Authors:** Erik Christensen, Ping Luo, Andrei Turinsky, Mia Husić, Alaina Mahalanabis, Alaine Naidas, Juan Javier Diaz-Mejia, Michael Brudno, Trevor Pugh, Arun Ramani, Parisa Shooshtari

**Affiliations:** Department of Computer Science, University of Western Ontario, London, ON, Canada; Children’s Health Research Institute, Lawson Research Institute, London, ON, Canada; Princess Margaret Cancer Centre, University Health Network, Toronto, ON, Canada; Centre for Computational Medicine, The Hospital for Sick Children, Toronto, ON, Canada; Centre for Computational Medicine, The Hospital for Sick Children, Toronto, ON, Canada; Centre for Computational Medicine, The Hospital for Sick Children, Toronto, ON, Canada; Children’s Health Research Institute, Lawson Research Institute, London, ON, Canada; Department of Pathology and Lab Medicine, University of Western Ontario, London, ON, Canada; Princess Margaret Cancer Centre, University Health Network, Toronto, ON, Canada; Department of Computer Science, University of Toronto, Toronto, ON, Canada; Princess Margaret Cancer Centre, University Health Network, Toronto, ON, Canada; Ontario Institute for Cancer Research, Toronto, ON, Canada; Department of Medical Biophysics, University of Toronto, Toronto, ON, Canada; Centre for Computational Medicine, The Hospital for Sick Children, Toronto, ON, Canada; Department of Computer Science, University of Western Ontario, London, ON, Canada; Children’s Health Research Institute, Lawson Research Institute, London, ON, Canada; Department of Pathology and Lab Medicine, University of Western Ontario, London, ON, Canada; Ontario Institute for Cancer Research, Toronto, ON, Canada

**Keywords:** single-cell RNA-seq, cancer, labelling, machine learning, automated algorithms

## Abstract

Single-cell RNA sequencing (scRNA-seq) clustering and labelling methods are used to determine precise cellular composition of tissue samples. Automated labelling methods rely on either unsupervised, cluster-based approaches or supervised, cell-based approaches to identify cell types. The high complexity of cancer poses a unique challenge, as tumor microenvironments are often composed of diverse cell subpopulations with unique functional effects that may lead to disease progression, metastasis and treatment resistance. Here, we assess 17 cell-based and 9 cluster-based scRNA-seq labelling algorithms using 8 cancer datasets, providing a comprehensive large-scale assessment of such methods in a cancer-specific context. Using several performance metrics, we show that cell-based methods generally achieved higher performance and were faster compared to cluster-based methods. Cluster-based methods more successfully labelled non-malignant cell types, likely because of a lack of gene signatures for relevant malignant cell subpopulations. Larger cell numbers present in some cell types in training data positively impacted prediction scores for cell-based methods. Finally, we examined which methods performed favorably when trained and tested on separate patient cohorts in scenarios similar to clinical applications, and which were able to accurately label particularly small or under-represented cell populations in the given datasets. We conclude that scPred and SVM show the best overall performances with cancer-specific data and provide further suggestions for algorithm selection. Our analysis pipeline for assessing the performance of cell type labelling algorithms is available in https://github.com/shooshtarilab/scRNAseq-Automated-Cell-Type-Labelling.

## Introduction

Single cell RNA sequencing (scRNA-seq) allows researchers and clinicians to precisely define complex cell populations by examining gene expression in thousands of individual cells per analysis. This is particularly useful in the context of cancer, as tumors and their surrounding microenvironment can be incredibly heterogeneous, consisting of various cell types including but not limited to malignant cells, cells of the tissue of origin, immune and endothelial cells and fibroblasts [1–4]. Such heterogeneity often has functional effects that lead to cancer progression, metastasis and treatment resistance [2, 5, 6], emphasizing the importance of correctly describing the cell types present in a given tumor. Recently, scRNA-seq technologies have been successfully used to elucidate tumor composition and identify specific cell types that negatively impact treatment response or that may be viable for immunotherapeutic targeting [4, 7, 8]. Use of scRNA-seq can thus provide critical insight into patient prognosis and guide the development of treatment options, driving the recent increase in implementation of such technologies in cancer research.

While most components of standard scRNA-seq pipelines are automated, cell type labelling requires researchers to manually examine the expression of marker genes in their data in order to annotate cell types. This often involves literature searches instead of standardized gene profile ontologies, is time-consuming, and can result in inconsistent nomenclature or labelling practices, making reproducibility and comparison across studies a challenge. To address these problems, several automated cluster labelling algorithms have been recently developed, typically implementing either unsupervised, cluster-based methods or supervised, cell-based methods [9, 10].

To assign labels to clusters, cluster-based methods first group cells based on their gene expression profiles, then assign cell type labels to each of these groups, or clusters. To do this, they make comparisons between the scRNA-seq dataset of interest and reference gene expression signatures, which are typically composed of marker gene sets compiled from literature searches or continuous marker gene expression values from experimental techniques such as microarrays or RNAseq [11, 12]. While these methods perform well, they are limited by their reliance on marker gene data, which is often not available for tissues of interest [13].

In contrast, supervised cell-based labelling approaches rely on training using annotated training datasets containing the same cell populations as the query data. Such algorithms make the assumption that gene expression patterns are discriminative between cell types, as this has been demonstrated in experiments using healthy tissue data [9]. Cell-based algorithms thus learn the distribution of each cell type from the annotated training dataset by training supervised models, which are then used to predict cell identities. If a query dataset, however, contains cell types not present in the reference data, cell-based algorithms cannot predict the identities of the corresponding cells. The accuracy of such methods may also vary with particularly heterogeneous cell groups [10], and computational times can be high [9].

Some methods do have functions created with cancer cells in mind [14]; however, most labelling tools were developed either outside of cancer contexts or for specific cell or tissue types, making it difficult to evaluate how well they may perform with highly diverse cancer datasets. Indeed, performance of scRNA-seq cluster labelling algorithms has been shown to decrease as the complexity and number of cell populations in a dataset increase [9]. With respect to cell-based algorithms, while gene expression patterns may be discriminative between healthy cell types, the same may not be necessarily true for tumors and their microenvironment [15]. This suggests that particular care must be taken when selecting an appropriate method for the labelling of cancer scRNA-seq data, as stem cell differentiation, accumulation of mutations and selective pressures from the tumor microenvironment can all drive tumor heterogeneity, increasing the number and complexity of cell subpopulations present in a given tumor sample. While comparisons between scRNA-seq cluster labelling algorithms have been previously made [9, 13, 16, 17], these evaluations focused on normal tissue samples and how well these tools might perform with more complex tissues such as tumors remains unclear.

In this study, 26 scRNA-seq cell type labelling algorithms are assessed to determine their performance in the specific context of cancer datasets. Nineteen of the algorithms are cell-based, requiring training datasets, while the remaining seven are cluster-based, and require marker gene sets. The performance of each algorithm was determined using eight cancer datasets, including brain, breast, lung, colorectal, leukemia, melanoma and pancreatic cancers. For cell-based methods, 5-fold cross-validation was used to evaluate the ability of a method to predict cell type identities. For cluster-based algorithms, clustering the cells followed by automated labeling was assessed. We evaluated the performance of each algorithm, taking into account several aspects and conditions that are of particular interest when working with cancer data. This includes assessing performance of the algorithms in detecting under-represented cell populations or specific categories of cell types, effects of imbalanced datasets on cell type predictions, as well as patient-based analyses. This study encompasses the largest cohort of cell type labelling algorithms assessed to date, and uses the largest cancer-focused test set to do this assessment. Our results exemplify the specific challenges associated with labelling of tumor scRNA-seq data, and highlight, which algorithms perform well in a cancer context, helping cancer researchers and clinicians select the preferred tools for their analyses.

## Results

**Figure 1:**
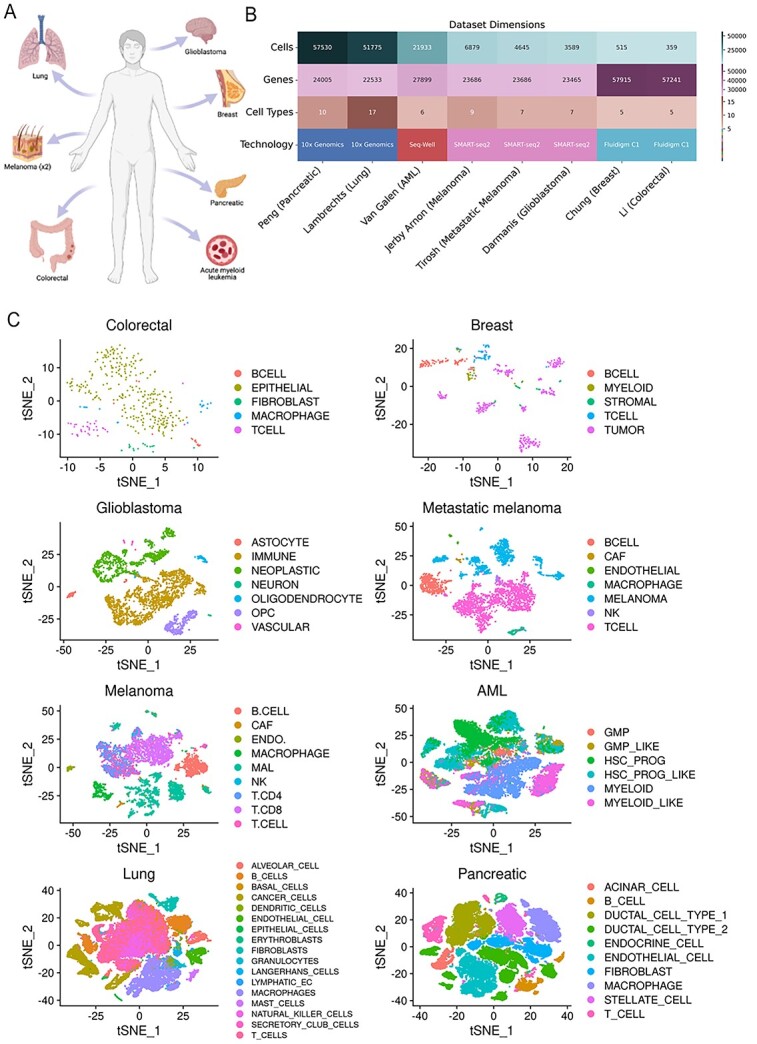
The eight selected cancer datasets provide a wide range of cell, gene and cell type numbers. (**A**) Eight cancer datasets from a variety of human tissues, generated using multiple sequencing technologies, were used in this study. (**B**) All numbers shown are after preprocessing has been done, and represent the size of the datasets given as input to all algorithms. (**C**) tSNE plot of each dataset colored by truth labels.

In this study, we examined the performance of 26 scRNAseq labelling algorithms using 8 scRNAseq cancer datasets. Our datasets span a variety of cancer types and are of varying sizes with respect to number of cells, genes and cell types, and were obtained using varying sequencing technologies (Figure 1). For all of these datasets, we obtained the truth labels from the original paper published the dataset. Figure 1C depicts tSNE plot colored by the truth labels for each dataset. Of the 26 algorithms we used, 7 use cluster-based approaches, while 19 are cell based. We also take into consideration several types of analyses and experimental conditions that are particularly relevant in cancer research and clinical application to provide the most thorough evaluation of labelling methods in a cancer context. This includes the detection of under-represented cell populations in specific datasets, correct labelling of cells from individual patients, and the effects of imbalanced data on labelling accuracy.

### Overall performance and running time of all algorithms

**Figure 2:**
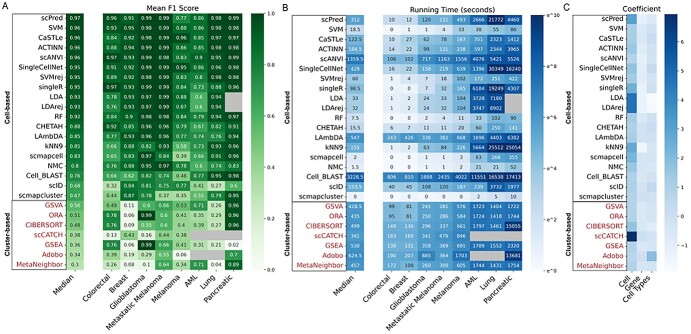
Performance and running time summary of cell type prediction methods across eight cancer datasets. (**A**) F1-scores of 19 cell-based methods (*Y*-axis, black labels) and 7 cluster-based methods (*Y*-axis, red labels) on 8 cancer datasets (*X*-axis). Datasets are sorted from fewest to most cells, and algorithms are sorted along the *Y*-axis according to their median F1 score across all datasets. For cluster-based algorithms, Seurat was used for clustering, and it was followed by labeling. Values are the median of a bootstrapped 95% confidence interval generated by sampling the predictions 10 000 times. Gray squares are those where the algorithm was unable to obtain a prediction due to scalability issues related to large sample sizes. Cell-based algorithms outperformed cluster-based algorithms, likely due to a lack of available gene signature data - particularly for malignant cells - that cluster-based algorithms rely on for accurate labelling. (**B**) Running time was calculated as ln(second + 1) for each dataset, and the algorithms were ordered based on their median F1 score across all datasets, shown in (**A**). Cell-based and cluster-based methods are again indicated using black and red labels, respectively, on the *Y*-axis. (**C**) Linear regression was performed based on the column normalized running time of the algorithms and the corresponding number of cells, genes and cell types in each dataset. Values in the heatmap denote the coefficients of the learned models.

We began by comparing the performance of each algorithm across all datasets, using F1 score as a performance metric. For each algorithm, we calculated F1 scores per individual dataset, as well as a median F1 score across all datasets to indicate overall performance (Figures 2A and3). Although some cluster-based algorithms performed well on specific datasets, median F1 scores indicate that cell-based algorithms outperform cluster-based algorithms in general. Overall, 11 cell-based algorithms showed an average F1 score of 0.9 or higher. The top performers were scPred, with an F1 score of 0.97, followed by CaSTLe, scANVI and SVM, with F1 scores of 0.96. In contrast, the most successful cluster-based algorithms were ORA, GSVA and CIBERSORT, with average F1 scores of 0.56, 0.51 and 0.48, respectively. We repeated this analysis using ARI, homogeneity, precision and recall as performance metrics and observed similar trends (Supplementary Figure S1).

**Figure 3:**
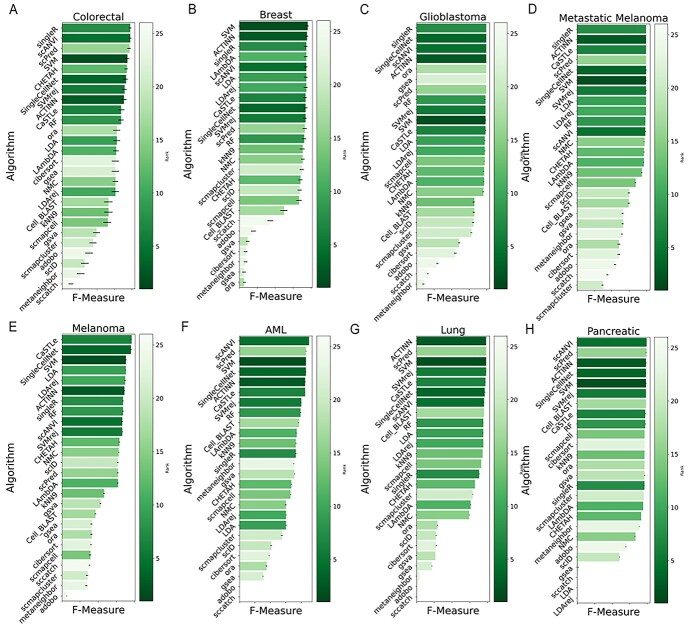
F1 Scores of the algorithms on each dataset. Barplots of F1 scores for each algorithm on (**A**) Breast cancer, (**B**) Colorectal cancer, (**C**) Glioblastoma, (**D**) Metastatic Melanoma, (**E**) Melanoma, (**F**) AML, (**G**) Lung cancer and (**H**) Pancreatic cancer datasets are presented. Black bars around each bar represent a 95% confidence interval. Each bar that corresponds to any given algorithm is colored based on the algorithm’s overall rank for the dataset of interest.

**Figure 4:**
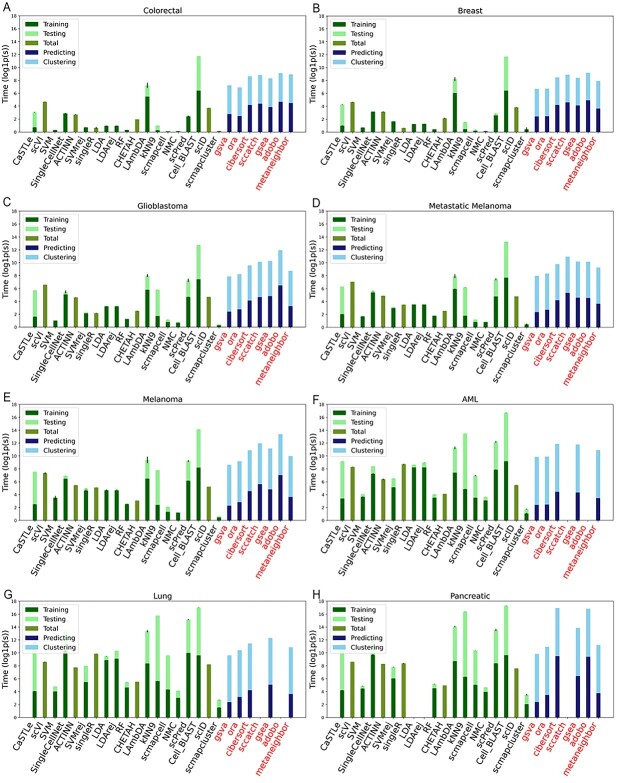
Detailing timing information for each algorithm on all datasets. The processing time of different algorithms for (**A**) Colorectal, (**B**) Breast, (**C**) Glioblastoma, (**D**) Metastatic Melanoma, (**E**) Melanoma, (**F**) AML, (**G**) Lung and (**H**) Pancreatic cancer datasets are shown. Bars are split into training and testing time or clustering and labelling time where possible. For algorithms that include separate training and testing steps, those times are shown as separate colors in the bar. For cluster-based algorithms, the time required to cluster the data is also shown.

To evaluate the speed and scalability of the 26 algorithms, we recorded and analyzed the running time of each algorithm on each of the 8 cancer datasets. Our calculations of the running time for cell-based algorithms consisted of training time and predicting (testing) time used in the cross-validation, while the running time of cluster-based algorithms consisted of time used for clustering and labelling. All the algorithms were running on high-performance clusters with 2 CPU cores (2500 MHz) and 8–120 GB RAM depending on the size of the datasets (see Methods). Figure 2B shows the results of the overall running time in ln(second + 1), with algorithms ordered based on their median F1 scores across the eight datasets. Figure 4 shows the training and testing or clustering and cluster labelling time for cell-based and cluster-based algorithms on all datasets, respectively. It should be noted that some cell-based algorithms implement training and predicting in a single function in their original published packages, thus we could only record the total running time for those methods. Our results show that cell-based algorithms were faster than cluster-based algorithms on smaller datasets (<10 000 cells) due to the time that clustering takes. This is in part due to normalization and other early processing steps in the Seurat pipeline. Furthermore, most algorithms require substantially more time for larger datasets (>20 000 cells), including AML, Lung and Pancreatic datasets. Of note, scPred, SingleR, CaSTLe, ACTINN, SingleCellNet, KNN 9, Cell_BLAST and scVI all have drastically longer runtimes (up to 200× longer) with larger datasets, such as lung and pancreatic, compared to their results with smaller datasets such as colorectal and breast (Figure 2B).

As our datasets vary in terms of numbers of cells, genes and cell types, we have evaluated the influence of these properties on running time (Supplementary Figure S2). Specifically, for each algorithm, a linear regression model was trained using the numbers of cells, genes and cell types as features, and the corresponding running time (ln(1 + second)) as targets. Then, the coefficients of the learned models were shown in Figure 2C. A higher coefficient means the algorithm’s running time is more affected by the corresponding feature, while a coefficient close to zero would represent the runtime of the algorithm would not change no matter how large or small the corresponding data size parameter would be. Of note, most correlation coefficients related to the number of genes and number of cell types were close to zero, while majority of the correlation coefficients related to the number of cells were non-zeros. This indicates that the majority of algorithms were primarily impacted by the total number of cells in a given dataset. An exception is Adobo, which is mainly impacted by the number of cell types. Our results also indicates that for all of the algorithms that we examined, the running time is not impacted by the number of genes (Figure 2C).

### Assessing performance of algorithms in detecting specific cell types

To further characterize the differences in performance between cell-based and cluster-based labelling algorithms, we compared the F1 scores of the algorithms per individual cell type in each of the eight datasets (Supplementary Figure S3). Our results again showed overall lower performance for the cluster-based methods compared to cell-based algorithms. Of note was a significant reduction in F1 scores on malignant cell types predicted by cluster-based algorithms. This is likely due to a lack of gene signatures for malignant cell types, which are required by most cluster-based prediction methods. In addition, we observed that scID and scmapcluster showed inconsistent performance on different types of cells. Although they are cell-based algorithms, their prediction relies on clustering analysis of the datasets, and the quality of the clustering cannot be guaranteed when using default parameters.

### Cell type and category analysis

**Figure 5:**
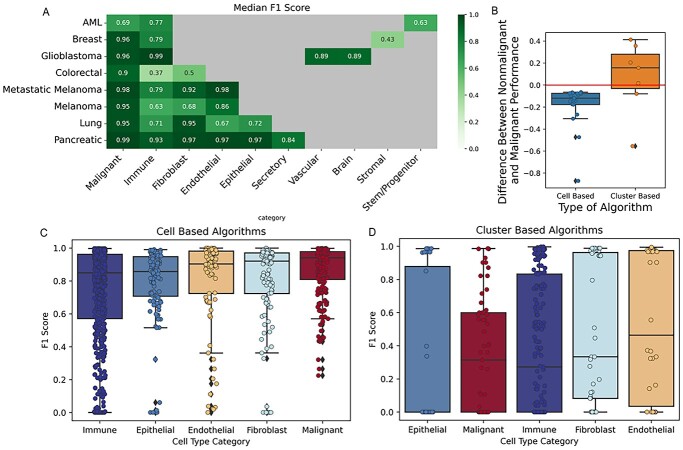
Cell type and category analysis. (**A**) Ten cell categories were identified across all eight datasets. A cell category score for any given algorithm on a dataset is the median of the per-class scores for that algorithm on the classes belonging to the category (i.e. the immune cell category score is the median performance on T Cells, B Cells and other immune-related cells). Datasets included in each category are: Malignant (all datasets), Immune (all datasets), Fibroblast (colorectal, metastatic melanoma, melanoma, lung, pancreatic), Endothelial (metastatic melanoma, melanoma, lung, pancreatic), Epithelial (lung, pancreatic), Vascular and Brain (glioblastoma), Stromal (breast) and Progenitor and Stem Cells (AML). (**B**) A boxplot showing the difference between median performance of nonmalignant cells and median performance of malignant cells. Results indicate that cluster-based algorithms performed better on non-malignant cells, whereas cell-based algorithms performed better on malignant cells. Boxplots show the F1-scores of (**C**) cell-based and (**D**) cluster-based algorithms on datasets for the eight most commonly seen cell type categories. Outliers in **C** are small cell populations predicted by algorithms that are unable to identify under-represented cell types (see Figure 8).

Accounting for all 8 datasets, we identified 10 cell categories including malignant, immune, fibroblast, endothelial, epithelial, secretory, vascular, brain, stromal and stem/progenitor cells using Cell Ontrology (https://www.ebi.ac.uk/ols/ontologies/cl) (Figure 5A). Of these, five categories were present in at least two datasets: malignant (eight datasets), immune (eight datasets), fibroblasts (five datasets), endothelial (four datasets) and epithelial (two datasets). We ranked how well these categories were labelled by the cell-based and cluster-based algorithms using the median F1 scores (see section Methods and Figure 5). For cell-based methods, highest performance was observed with malignant cells, followed by fibroblasts, endothelial, epithelial and finally immune cells (Figure 5C). Cluster-based methods showed the highest performance with endothelial cells, followed by fibroblasts, immune, malignant and epithelial cells. Of note, the median scores obtained across these five categories with cluster-based algorithms had a much greater spread compared to the median scores obtained with cell-based algorithms (Figure 5C and D). This may indicate that performance varies more across different algorithms and datasets depending on the quality of gene signatures and the prediction method. In scRNA-seq labelling applications, it is preferable to use algorithms that have higher performance with lower variabilities across different cell-type categories. Our results indicate that cell-based algorithms (Figure 5C) may be preferred over the cluster-based algorithms (Figure 5D) for that reason.

Further analysis of algorithm performance on malignant and non-malignant cell types using a Wilcoxon rank-sums test showed a significant difference between the cell-based and cluster-based methods (Figure 5B). Specifically, for cell-based methods, the highest performance was observed for malignant cells, whereas for cluster-based methods malignant cells were often completely unidentifiable.

### Evaluation of cell-based algorithm performance

Examining the performance of supervised cell-based algorithms, we found that they were much more successful at correctly labelling malignant cells compared to non-malignant cell types, as indicated by a Wilcoxon rank sums test (*P* < 0.05) (Figure 5B). To see if this was due to malignant cell populations simply providing more training data, we measured for each dataset the correlation between malignant cell proportion and F1 scores, as well as the correlation of non-malignant cell proportion and F1 scores. We observed no correlation between the proportion of malignant cells in a dataset and an algorithm’s ability to identify malignant cells (Supplementary Figure S4).

We did however observe that the proportion of any given cell type within a dataset appears to affect prediction scores for that cell type in a non-linear fashion. Specifically, we found that Spearman’s correlation shows a slight correlation between proportion of cells and an algorithms prediction score on that cell type for the glioblastoma (rho = 0.64, *P* = 1.27e − 17) and breast cancer (rho = 0.86, *P* = 5.25e − 32) datasets (Supplementary Figure S4). This may be due to higher numbers of malignant and immune cells relative to other cells of the tissue of origin in many of the datasets, and drove us to examine the effects on cell prediction with balanced datasets, in which each cell type is equally represented.

### Examining the stability of algorithm performance on imbalanced datasets

**Figure 6:**
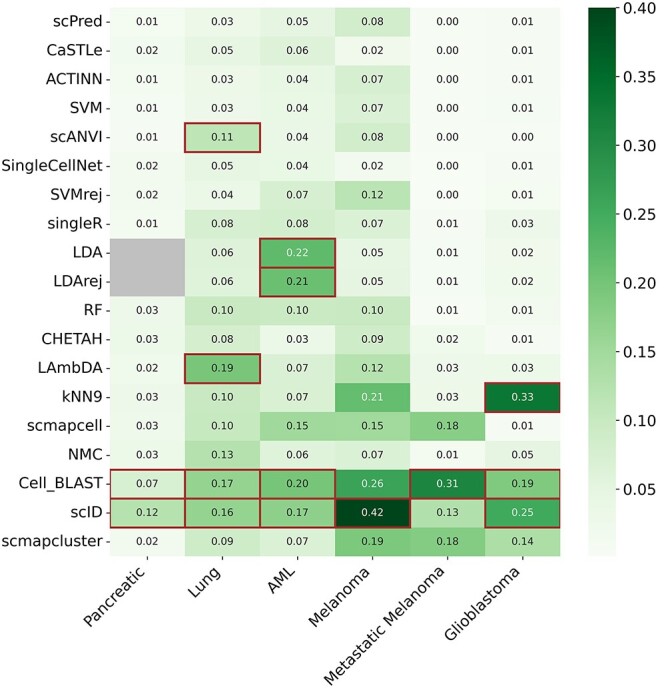
Algorithm performance across subsamples of datasets. The heatmap shows the mean absolute value change in F1 scores (between the balanced data after sub-sampling and the original data) on all cell types for each algorithm and dataset. We then used hierarchical clustering to identify unstable algorithms for each dataset. Singletons are indicated using a red outline.

Most scRNAseq datasets have multiple cell type populations of varying sizes, creating an imbalance in terms of cell population frequencies (Supplementary Figure S5). To expand upon the above analysis, we examined the stability of each labelling algorithm, or its ability to consistently identify all cell types in a given dataset, regardless of substantial differences in the number of cells present per each type. We first subsampled 800 cells (Supplementary Figure S6) with replacement from each cell type in each of our six largest datasets (pancreatic, lung, AML, melanoma, metastatic melanoma and glioblastoma) to generate balanced datasets in terms of frequency of cells across cell types. We then measured the F1 scores of cell-based algorithms with the subsampled data. Subsample F1 scores were compared to initial F1 scores using full-sized datasets to identify algorithms that exhibit the largest changes in performance when more training data is available. Large differences between a specific algorithm’s F1 scores using a full dataset and its subsampled counterpart were considered to indicate a strong effect of imbalanced data on performance, suggesting algorithm instability (Figure 6).

Our results showed that most algorithms see substantial changes on at most one dataset. Algorithms with substantial F1 score fluctuations in five of the six datasets were considered unstable, and included CellBLAST and scID. CellBLAST is an autoencoder-based method, which learns latent representation for cell type prediction. To reduce false positive predictions, the algorithm trains multiple models with different starting points and predicts cell type labels with each of them. A significance score is computed for every prediction in each model. If a prediction is not significant in a majority of the models, the corresponding result would be rejected. If reference datasets are relatively small, models may not be trained sufficiently, potentially resulting in insignificant—and thus rejected—predictions like what we observed with this analysis. scID learns gene signatures for each cell type from the reference dataset and uses them by a linear discriminant analysis model to predict the query cell type labels. With a smaller reference dataset, the quality of the learned gene signatures may affect the performance of the algorithm.

### Performance when training and testing on separate patients

Typical 5-fold cross validation trains a classifier on a randomly selected subset of cells from all patients. This may not reflect a real-world situation, where the classifier will predict cell types in a new patient with potentially different tumor microenvironment cell composition from the patients used for training. In order to simulate this scenario, we created training and testing sets composed of entirely different patients. We found that training algorithms on one subset of patients and testing on another led to accurate identification of cells by 14/19 cell-based algorithms; (Table 1; last column).

**Table 1:** A summary of performance of all algorithms

Algorithm	Median F1 score	Median run time (s)	Stable for imbalanced data	Identifies under-represented cell types	Identifies cells when training on different patients
scPred	0.97	312.0	Yes	Yes	Yes
CaSTLe	0.96	122.5	Yes	Not in extreme cases	Yes
scANVI	0.96	1359.5	Yes	No	Yes
SVM	0.96	18.5	Yes	Yes	Yes
ACTINN	0.95	184.5	Yes	Not in extreme cases	Yes
SingleCellNet	0.95	429.0	Yes	Not in extreme cases	Yes
SVMrej	0.94	60	Yes	Not in extreme cases	Yes
singleR	0.94	98.5	Yes	Yes	Yes
LDArej	0.91	32	Yes	Not in extreme cases	Yes
LDA	0.91	33	Yes	Not in extreme cases	Yes
RF	0.91	7.5	Yes	No	Yes
CHETAH	0.88	15.5	Yes	Not in extreme cases	Yes
LAmbDA	0.85	547	Yes	Not in extreme cases	Yes
kNN9	0.85	155	Yes	No	No
scmapcell	0.83	2	Yes	No	Yes
NMC	0.8	1.5	Yes	Not in extreme cases	No
Cell_BLAST	0.76	3228.5	No	No	No
scID	0.68	153.5	No	No	No
scmapcluster	0.67	<1	Yes	No	No
GSVA	0.56	428.5	–	No	–
ORA	0.51	435	–	No	–
CIBERSORT	0.48	499	–	No	–
scCATCH	0.38	341	–	No	–
GSEA	0.36	530	–	No	–
Adobo	0.34	624.5	–	No	–
MetaNeighbor	0.30	457	–	No	–

*Note:* Algorithms are assessed based on multiple criteria including their F1 scores, running times, whether they are stable for imbalanced data, whether they can identify under-represented cell types, and how they perform when trained and tested on different patients. Here, the blue, orange and pink colours indicate high, medium and low performance, respectively.

**Figure 7:**
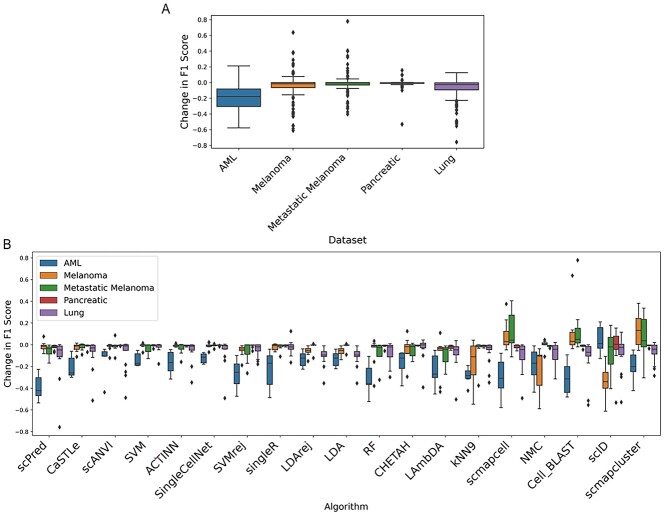
Boxplots showing the change in F1 score when algorithms are trained on groups created by separating samples from entire patients instead of randomly separating cells from all patients. (**A**) Change in F1 score of all classes for all algorithms in the five datasets. (**B**) Change in F1 score of all classes in each dataset for all algorithms.

Most algorithms see very small changes to the per-class F1 scores of the majority of the datasets (Figure 7B), which suggests that these algorithms can be used in clinical settings and are suited to many cancers, but not necessarily all.

Larger changes were, however, observed with the AML dataset, irrespective of which algorithm is chosen. Specifically, the large negative changes were evident for AML data in all the algorithms that are able to identify cells when training and testing on different patients for other datasets (Figure 7A). AML dataset examines stem cells across several timepoints in multiple patients, which may affect algorithm ability to identify cells this way.

### Under-represented cell type detection

**Figure 8:**
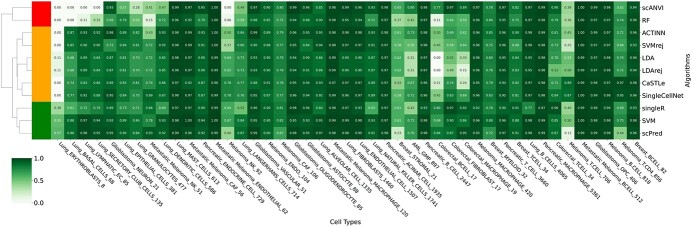
A heatmap showing the F1 scores of the top 11 algorithms (median F1 scores >0.9) on the under-represented cell types in all datasets. We defined under-represented cell types as those that are either fewer than 1000 cells and less than 30% of the entire dataset, or those that are less than 5% of the entire dataset. Algorithms are clustered using hierarchical clustering. As shown by the dendrogram on the left side of the figure, there are three groups of algorithms in terms of their capability in detecting under-represented cell types: those which are successful in detecting under-represented cell types (green); those which are not successful in detecting extremely under-represented cell types (yellow); and those which are not successful in detecting under-represented cell types (red).

Some scRNA-seq datasets have cell types that represent only a small fraction of the complete sample. These cell types may be hard for some algorithms to recognize due to the low amount of reference cells available to learn from in the training data. To examine the ability of our algorithms in detecting under-represented cell types, we first considered cell types representing either less than 5% of a dataset or fewer than 1000 cells and less than 30% of a dataset. We then examined the F1 scores of all algorithms on these cell types, using hierarchical clustering, which revealed three algorithm groups (Figure 8, Supplementary Figure S7). scPred, SVM and SingleR were always able to identify under-represented cell types. CaSTLe, ACTINN, SingleCellNet, SVMrej, LDA, LDArej, CHETAH, LAmbDA and NMC were unable to identify only the extremely under-represented cell types present, and the remaining 14 algorithms were unable to identify under-represented cell types.

### Algorithm recommendations

We assessed the performance of all algorithms in terms of their overall F1 score, running time, their capability in detecting under-represented cell populations, their stability in imbalanced datasets, and their performance when trained and tested on different patient subsets. A summary of these comparisons is provided in Table 1. Overall, scPred and SVM are the top-performing algorithms. Both have very high F1 scores (0.97 and 0.96, respectively) (Figure 2A), and are able to identify under-represented cell types (i.e. cell types with <5% of all cells, and cell types with <1000 cells comprising less than 30% of all cells) (Figure 8). In addition, both scPred and SVM are reasonably fast (Figure 2B), though SVM is 16 times faster than scPred when considering median running time across all datasets (18.5 versus 312.0 in seconds). CaSTLe, ACTINN and scANVI are also very strong performers (F1 score: 0.95) (Figure 2A). However, all three are at least six times slower than SVM with median running times of 122.5, 1359.5 and 184.5 in seconds compared to 18.5 s for SVM (Figure 2B). In addition, scANVI is unable to identify several under-represented cell types (Figure 8), and its F1 score drops to 0.75 when averaged across under-represented cell types.

Overall, 11 algorithms had F1 scores above 0.9: ACTINN, CaSTLe, LDA, LDA + rej, RF, scANVI, SingleCellNet, SingleR, scPred, SVM and SVM + rej. All are cell based. They had the 11 highest F1 scores (Figure 2), providing optimal performance, and all showed stable performance when using imbalanced datasets (Figure 6). Importantly, they maintained consistent performance when training and testing on separate patients, suggesting they would be valid in a clinical setting where classifiers trained on previously sampled patients are used to predict cell types in new patients.

It should, however, be noted that while singleR, SVM and scPred are able to identify all under-represented cell types, scANVI and RF are unable to identify under-represented cell types, and 6 other algorithms within the top 11 are unable to identify the extremely under-represented cells present. Furthermore, scANVI, SingleCellNet and singleR had particularly long run times with larger datasets such as the lung and pancreatic datasets.

## Discussion

We present here an evaluation of 26 scRNAseq labelling algorithms using 8 scRNAseq cancer datasets in what is the largest and most thorough cancer-specific scRNA-seq labelling method benchmarking study. To determine the performance of each algorithm using varied inputs, we chose datasets with diverse features, including cell numbers spanning from 539 to over 57,000, a range of gene numbers, and as many as 17 different cell types. Meanwhile, to minimize the study design bias, the relationship between the 8 datasets and the 26 algorithms were investigated (Supplementary Tables S1 and S2), and results showed that only the AML dataset was labeled by RF, which would not have a huge impact on our evaluation. We evaluated the performance of the algorithms for each dataset overall as well as within specific experimental conditions of interest to cancer research, thus giving a more complete representation of how well they are able to label cancer datasets.

Our work showed that cell-based algorithms largely outperformed cluster-based algorithms, with CaSTLe, scANVI and SVM showing the best overall performance among cell-based methods, while ORA, GSVA and CIBERSORT were the highest performing cluster-based methods. Of note, two of the top performing algorithms (SVM and scPred) were support vector machine (SVM)-based methods. Cell-based methods also showed faster running times, particularly on smaller datasets, due to normalization and additional preprocessing required by cluster-based methods.

In addition, cell-based algorithms showed particularly high performance on malignant cells in cell type and category-based analyses. For other, less abundant cell types, balanced data analyses showed that lower scores are driven by a lack of training data. Similarly, overrepresentation of certain cell types in full datasets appeared to result in higher performance. Thus, our findings indicate that training data availability can significantly impact performance of certain algorithms identified as unstable.

It is, however, interesting to note that particularly unique cell types may require a smaller number of cells for training data purposes. In the Lung dataset, we see this with mast cells, fibroblasts and endothelial cells; algorithm performance in the original dataset is high despite these cell types being underrepresented, and no significant increase in the balanced dataset was observed. This may be because these cell types are different enough from other cell types in the dataset, making them distinguishable easily by the algorithms.

As indicated by category-based analyses, cluster-based methods showed poor labelling of malignant cells compared to cell-based algorithms. This is likely due to a lack of high-quality reference gene signatures available for these cell subpopulations, as many cluster-based algorithms rely on reference gene signatures to accurately predict cell type labels. They did, however, perform better with non-malignant cells compared to malignant cells. They also have the benefit of not requiring additional training data, making them the method of choice for many users. Nonetheless, they were largely outperformed by cell-based algorithms, making the development of improved cluster-based scRNA-seq labelling algorithms desirable. It should be noted that the number of cells in a dataset had a substantial impact on running time for both types of algorithms, and should be taken into consideration when selecting a labelling method or developing new algorithms. To be more specific, cell-based algorithms usually need more time to train their models when the dataset contains more than 20 k cells. On the other hand, cluster-based algorithms need more time to finish the clustering analysis as the number of cells increase. Only a few algorithms, such as SVM, RF and CHETAH, can achieve consistent high running speed on all eight datasets.

Our study is the first to examine the ability of scRNAseq labelling algorithms to not only identify cells within the tumor microenvironment, but also accurately predict cell types under a variety of conditions. Such in-depth evaluations are critical for fully understanding the applicability of such algorithms to cancer data. Specifically, we show that some algorithms can identify common cell types within a dataset, but fail to identify less common or under-represented cell types (Figures 6 and 8). Identifying all cell subpopulations is particularly important when working with cancer patient data, as this can guide treatment selection and prevent treatment resistance and disease recurrence. We also show that many algorithms are able to accurately predict cell types when trained and tested on different patients, despite potentially drastic differences in the tumor landscape from patient to patient. This shows that supervised learning algorithms can eventually be used in a clinical setting to accurately characterize cell compositions in patient tumor samples.

It should be noted that most algorithms evaluated in our study have their own implementations, and learning and incorporating these methods into a normal single cell analysis pipeline would be time-consuming. To help researchers better use the top performing algorithms and integrate their results with other single cell analysis, we plan to implement them into our online platform CReSCENT (CanceR Single Cell ExpressioN Toolkit, crescent.cloud) [18]. CReSCENT is a web portal incorporating a series of pipelines for standardized analysis of scRNA-seq data. It also includes many public cancer datasets, which could be used as reference in the cell-based labeling. Currently, we have implemented GSVA on CReSCENT, and SVM is under development. Other top performers identified in this study, such as CaSTLe and scANVI, will also be implemented in the future.

## Methods

### scRNA-seq labelling algorithms

For this study, we compared a total of 26 scRNA-seq cell type-labelling algorithms. Of these, 19 rely on cell-based, supervised methods, including 7 general purpose algorithms not developed specifically for use with scRNA-seq. The seven remaining algorithms are unsupervised cluster-based methods. Our cohort represents a wide variety of approaches used for cell type labelling, and encompasses those developed specifically for scRNA-seq data as well as general-purpose tools frequently implemented in scRNA-seq analyses. This allowed us to thoroughly evaluate a broad set of strategies for scRNA-seq cluster labelling to determine which tools perform favorably when analyzing tumor samples. Names, categories, availability and descriptions of each algorithm are provided in Table 2. Here, we provide a brief description of various algorithms used in our study.

**Table 2:** Summary of cell-type labelling algorithms used

Name (source)	Category	Language	Brief description
ACTINN (Ma and Pellegrini 2020) [19]	Cell-based	Python	Neural network-based with three hidden layers
CaSTLe (Lieberman *et al*. 2018) [20]	Cell-based	R	XGBoost classification model with random forest classifier
Cell BLAST (Cao *et al*. 2020) [21]	Cell-based	Python	Modified neural network-based classifier; cell-to-cell similarity
CHETAH [14]	Cell-based	R	Hierarchical classification based on similarity to reference dataset
KNN 9 [22]	Cell-based	Python	General purpose *k*-nearest neighbor-based classifier, with *k* = 9
LAmbDA (Johnson *et al*. 2019) [23]	Cell-based	Python	Transfer learning based on *k*-nearest neighbor
LDA [22]	Cell-based	Python	General purpose classifier using linear discriminant analysis
LDA + rej [22]	Cell-based	Python	LDA with rejection option
NMC [22]	Cell-based	Python	General purpose classifier using nearest mean classification
RF [22]	Cell-based	Python	General purpose classifier using random forest method
scID (Boufea *et al*. 2020) [24]	Cell-based	R	Framework based on Fisher’s linear discriminant analysis
scmap-cell (Kiselev *et al*. 2018) [25]	Cell-based	R	Classifier based on distance between a cell and the nearest cell type
scmap-cluster (Kiselev *et al*. 2018) [25]	Cell-based	R	Classifier based on distance between a cell and the nearest cluster centroid
scPred (Alquicira-Hernandez *et al*. 2019) [26]	Cell-based	R	Support vector machine (SVM)-based classifier; combines dimension reduction with machine learning for classification
scANVI (Xu *et al*. 2021) [27]	Cell-based	Python	Deep generative model with Variational Inference
SingleCellNet (Tan and Cahan 2019) [28]	Cell-based	R	Random forest-based classifier
SingleR (Aran *et al*. 2019) [29]	Cell-based	R	Classifier based on correlation between reference and test data
SVM [22]	Cell-based	Python	General purpose classifier using a support vector machine method with linear kernel
SVM + rej [22]	Cell-based	Python	SVM with rejection option
adobo (Franzén and Björkegren 2020) [30]	Cluster-based	Python	Cluster labelling based on weighted marker gene expression and cell type activity scores
CIBERSORT [11]	Cluster-based	R and Java	Linear support vector regression-based labelling
GSEA (Subramanian *et al*. 2005) [31]	Cluster-based	Java	Determines gene expression patterns using weighted enrichment scores
GSVA [12]	Cluster-based	R	Determines gene expression patterns using discrete Poisson kernel-based enrichment scores
MetaNeighbor (Crow *et al*. 2018) [32]	Cluster-based	R	Cell type labelling based on neighbor voting
ORA (Goeman and Bühlmann 2007) [33]	Cluster-based	R	Cell type labelling based on over-representation of given gene sets
scCATCH [10]	Cluster-based	R	Cell type labelling with evidence-based scoring protocol

Cluster-based algorithms are mainly designed to automatize the traditional manual labeling process, which includes clustering cells into different groups and labeling cells in each group based on their marker genes. Different algorithms use different strategies to select marker genes and predict the cell identity with their unique gene signatures. More specifically, ORA and scCATCH [10] select marker genes based on differential expression; GSEA and adobo select marker genes based on gene expression levels; GSVA [12] use a sample distribution and expression level statistic to rank genes; and CIBERSORT used support vector regression to identify support vectors (maker genes).

Cell-based algorithms, on the contrary, use a reference dataset and label each cell based on its relationship with the reference. Typical examples are scmap (cell/cluster) and SingleR, which compute the similarities between the reference cells and the query cells to achieve prediction. Another type of cell-based algorithms train machine-learning models to learn cell-type-specific patterns from the reference dataset and use them to label query cells. This includes general purpose algorithms (LDA, KNN, NMC, RF, SVM, etc. [22]) and algorithms specifically designed for single cell data including scPred, CaSTLe, scANVI, ACTINN, SingleCellNet, SingleR, CHETAH, LambDA, Cell_BLAST and scID. General purpose algorithms are usually fast and easy to implement; however, they might suffer from overfitting, because many reference datasets contain much more genes (features) than cells (samples). As a result, some algorithms tried to perform dimensional reduction first on the expression matrix and then train the model with general purpose algorithms. For example, scPred first uses singular value decomposition to reduce the dimension of the dataset, then apply SVM to predict cell types. SingleCellNet uses top-pair transformation to turn the expression matrix into a binary matrix of selected genes and train random forest (RF) to predict cell types. ScID and CaSTLe perform feature selection first and then apply LDA and XGBoost classifier to predict cell types, respectively. In addition, with more and more reference datasets available, algorithms based on neural network (LambDA, ACTINN, Cell BLAST, scANVI) were developed, and models with different architectures were trained to predict cell types.

Overfitting could also be an issue for these algorithms when they are trained on small reference datasets. However, this issue should be solved when large reference datasets are available. To guarantee our evaluation is not affected by overfitting, 5-fold cross-validation was used in our experiments, and algorithms would not obtain a high overall F1 scores if they were overfitted on small datasets.

### Datasets

While many of the algorithms we selected were previously considered in studies comparing various scRNA-seq labelling methods, neither of these studies focused on cancer datasets specifically and instead primarily used normal tissue samples [9, 13]. Therefore, to evaluate how accurately these tools are able to label complex tumor data, we thoroughly searched existing public repositories and found appropriate cancer scRNA-seq datasets. We reasoned that to evaluate the performance of various algorithms, we need to access cell type labels from the original studies to benchmark the results of algorithms against them. Also, since several of the cluster-based algorithms required gene signatures, we particularly looked for datasets with available gene signatures. In addition, since most of the algorithms we evaluated required counts data as input, we picked the datasets with either available counts data or raw FASTQ files. Although several scRNA-seq datasets have been generated recently, only for a small portion of them (<20%), all the three (i.e. cell type labels, gene signatures and raw counts/FASTQ data) are available publicly. Our search resulted in the identification of eight datasets with these characteristics (Supplementary Table S3, and Figure 1B). The datasets could be obtained from either Gene Expression Omnibus (GEO), ArrayExpress (AE) or Genome Sequence Archive (GSA), and all of them are also accessible through the TMExplorer single-cell RNA-seq database [34].

#### Breast cancer

Single cells from 11 human primary breast cancer tumors are obtained from 11 patients. Four different subtypes comprise this dataset, including cancer cells, stromal cells and immune cells from the tumor microenvironment [35]. This dataset is available on the GEO portal under GSE75688.

#### Colorectal cancer

This dataset represents single cells isolated from 11 human primary colorectal cancer tumors from 11 patients at varying stage [36]. Cell types include T cells, B cells, macrophages, fibroblasts, mast cells, epithelial cells and malignant cells from the tumor microenvironment. This dataset is available on the GEO portal under GSE81861.

#### Glioblastoma

This dataset details the gene expression profiles of single cells isolated from four primary glioblastoma patients [37]. It consists of cells from the tumor core and peritumoral space of each patient, and samples are composed of malignant cells, vascular cells, immune cells, neuronal cells and glial cells. This dataset is available on the GEO portal under GSE84465.

#### Melanoma

This dataset contains gene expression profiles for single cells isolated from 33 human melanoma tumors (from 31 melanoma patients), 17 of which were newly collected from patients and 16 of which were from previously reported tumors [38]. Cell types include malignant cells, stromal cells and immune cells that compose the melanoma tumor microenvironment. This dataset is available on the GEO portal under GSE115978.

#### Metastatic melanoma

This dataset consists of single-cell expression profiles for cells isolated from 19 human melanoma tumors obtained from 19 melanoma patients with variety of clinical and therapeutic backgrounds [4]. It includes malignant, immune and stromal cells taken from ten metastases to lymphoid tissues, five metastases to subcutaneous or intramuscular tissue, three metastases to the gastrointestinal tract and one primary acral melanoma. This dataset is available on the GEO portal under GSE72056.

#### Lung

For this dataset, single-cell expression profiles were generated for cells isolated from five patients with untreated, non-metastatic lung squamous carcinoma or lung adenocarcinoma [39]. Cells were isolated from both tumor and normal lung tissue, and cell types present include cancer cells, immune cells, fibroblasts, endothelial cells, alveolar cells and epithelial cells. This dataset is available on the ArrayExpress portal under E-MTAB-6149, E-MTAB-6653.

#### Pancreatic

This dataset consists of pancreatic cells isolated from 11 control pancreases, and 24 primary pancreatic ductal adenocarcinoma tumors from 24 patients [40]. It comprises various subgroups of malignant and stromal cell types. This dataset is available on the GSA portal under CRA001160.

#### AML

This dataset is composed of cells from bone marrow aspirates from 16 AML patients and 5 healthy donors [41]. Cell types include hematopoietic stem cells, as well as populations of erythroid, lymphoid and myeloid cell types. This dataset only includes gene signatures for some of the total cells published, and any cell type without gene signatures available was removed for our study. This dataset is available on the GEO portal under GSE116256.

### Data preprocessing

We performed the following steps prior to the running of all cell type labelling algorithms: (i) collection of count data; (ii) removing cells with unknown truth labels; and (iii) normalization of counts. While data preprocessing specifics are unique to each method, we first collected raw count data from the TMExplorer database, which contains more than 27 scRNA-seq datasets from tumor microenvironments [34]. We selected these eight datasets from the database based on cell type label availability, which is necessary for evaluation of the performance of the cluster labelling algorithms. Seven out of the eight datasets have raw count data, while the Breast dataset only has TPM data. We downloaded the raw sequencing data for the breast cancer dataset, aligned it to the human genome (hg38) using the Galaxy pipeline, and obtained the count data [42].

### Cell type prediction

Cell-based algorithms take a matrix of individual cells’ gene expressions as input and return a prediction for each cell using supervised learning methods. Cluster-based methods first require cells to be grouped into clusters, then take the average gene expression in those clusters as input.

For cell-based methods with built-in normalization techniques, counts data were directly provided to the algorithms. For general purpose algorithms from the scikit-learn package [22] not developed specifically for scRNAseq data (Table 2), we calculated log10(count + 1) values for the *gene x cell* matrices of every dataset. We used 5-fold cross-validation to predict cell type labels. More specifically, each dataset was divided into 5-folds, with a stratified strategy such that each fold contains equal proportions of each cell type. We then used the same training and testing folds for all the methods. For general purpose algorithms with a rejection function, 0.7 was used as the threshold so that predictions with lower confidence were rejected and marked as ‘Unlabeled’. For other algorithms with a built-in rejection step, their default parameters were used to reject unconfident predictions.

For cluster-based methods, raw counts data were loaded into the Seurat v3.2 pipeline and normalized using Seurat’s *LogNormalise* method, after which we applied Seurat *Louvain* clustering with default parameters [43]. After clustering, we used Seurat’s output partitions to compute the average expression per cluster for cluster-labelling algorithms. For algorithms with built-in normalization techniques, we provided an average of raw count expression per cluster, and for algorithms without built-in normalization we normalized the raw counts using full quantile normalization prior to computing average expression per cluster. These average expressions per cluster are provided to cluster-based labelling algorithms (i.e. GSVA, GSEA, ORA, Adobo, scCATCH, CIBERSORT, MetaNeighbor) to obtain a set of predicted labels. These methods obtain predictions by comparing differentially expressed genes within each cluster to a list of genes that are known to be strongly expressed by certain cell types. GSVA, GSEA, ORA, CIBERSORT and MetaNeighbor take a list of cell type signature gene sets as input, while Adobo and scCATCH use their own built-in databases.

### Ranking cell type labelling algorithms

To evaluate and compare the ability of each algorithm to identify specific cells within the eight datasets, we used F1 scores, or the weighted harmonic mean of precision and recall. To calculate F1 scores, we first computed the score for each individual cell type according to the equation:

$$ F=2\times \frac{\mathrm{precision}\times \mathrm{recall}}{\mathrm{precision}+\mathrm{recall}} $$

In this formula, precision refers to the number of correctly labelled (true positive) cells out of the total number (false positive + true positive) of cells predicted to be that type in a given dataset, while recall refers to the number of cells correctly labelled as a given cell type out of all cells known to be that type (true positive + false negative) in a given dataset.

Since this is a multi-class problem, we cannot represent the performance of each algorithm on a dataset as a single score using the above formula. To obtain a single score for each algorithm on a specific dataset we used micro-averaging, where the final F1 score is the sum of the F1 scores for each cell type in a dataset weighted by the frequency of the cell type. In the following formula, *W_i_* is the frequency of a cell type, *F_i_* is the F1 score for a cell type and *n* is the number of cell types.

$$ \hat{F}={\sum}_{i=1}^n{W}_i{F}_i $$

In order to obtain a 95% confidence interval for the F1 scores, we used bootstrapping. Ten thousand re-sampled prediction sets were generated from a labelling algorithm output using subsampling with replacement, and an F1 score was calculated for each prediction set. The 50th percentile of these F1 scores represents the median F1 Score used for comparison, and the 2.5th and 97.5th percentiles determine a 95% confidence interval for an algorithm’s performance on a dataset (Figure 3).

In addition to the F1 score, we also explored four other measures of accuracy: precision, recall, adjusted Rand index (ARI) and homogeneity. To explore their degree of mutual redundancy, we first assembled a set of labelling partitions generated by different algorithms on different datasets. For each labelling partition, its five accuracy measures were computed and a principal component analysis was then performed on the set of partitions. All measures were highly correlated among each other as well as with the first principal component PC1, whereas the other principal components were correlated to a much lower extent with the five measures (Supplementary Figure S8). The analysis was done separately for cell-based and cluster-based algorithms and returned similar results. This suggested that the F1 score was an adequate representation of the group of quality measures that we examined; hence, we used the F1 scores in subsequent calculations.

### Resources used for evaluation

All the algorithms were running on high-performance clusters with two CPU cores (2500 MHz) and 8–120 GB RAM depending on the size of the datasets. The small size datasets (Breast and Colorectal) could successfully run with 8 GB RAM; while medium size datasets (Melanoma, Metastatic Melanoma and Glioblastoma) required 30 GB of RAM, and large datasets (Lung and Pancreatic) needed 120 GB of RAM. The same amount of RAM was used for all algorithms when running them on the same dataset, making our comparisons consistent across algorithms. The only exception was SingleCellNet, which required 300 GB RAM for successfully running on the Pancreatic and Lung datasets.

### Cell type and cell category specific analysis

To compare performance of each algorithm on various cell types, we collected the F1 scores for all cell types in each dataset and compared them across algorithms (Supplementary Figure S3). We also evaluated the performance across various cell type categories to assess whether certain algorithms are better at detecting certain cell categories (Supplementary Figure S9). For this, individual cell types in each dataset were first grouped into broader categories using the Cell Ontology (https://www.ebi.ac.uk/ols/ontologies/cl) [44]. Each category is composed of similar cell types based on their ontological relationships; for example, T-cells, B-cells and other immunological cell types would all be categorized as Immune cells.

For a given category, a median score was calculated using the per-class F1 score of each cell type within that category. Specifically, we first generated an F1 score for each possible combination of algorithm/dataset/cell type. Using this, we then calculated the median score of all cell types within a given category for each dataset to create F1 scores for each possible algorithm/dataset/category combination. We further compared the performances of all algorithms on labelling cell types designated as malignant (tumor) and those designated as non-malignant (non-tumor) using a Wilcoxon rank-sums test.

### Patient-based analysis

To compare each cell-based algorithm’s ability to correctly identify the potentially different cells in a new patient, we gathered data identifying which cells in each dataset belong to which patient. For this analysis, we included Melanoma, Metastatic Melanoma, Lung, Pancreatic and AML datasets, which had patient information available. We then separated the five datasets into 5-fold training and testing sets, making sure that each test set was composed of unique groups of patients. Using these new test sets, we re-trained, predicted the cell types and scored all 19 cell-based algorithms. This more closely simulates a real-world scenario where a lab may use a pre-trained classifier to identify the cells present in new patients.

### Analysis of performance on imbalanced datasets

We examined the performance of algorithms on imbalanced data, in which some cell types may contain many more samples than other cell types in the same dataset. We first generated synthetically balanced data by subsampling each dataset for 50, 100, 200, 400 or 800 cells per cell type present. Then we assessed changes in the performance of these algorithms when applied to synthetically ‘balanced’ data compared to the original ‘imbalanced’ data. As the Breast and Colorectal datasets are particularly small (564 and 376 total cells, respectively), we did not include them in this analysis. Thus, using 5 subsample sizes on 6 datasets provided us with 30 ‘balanced’ subsampled datasets. Cell-based algorithms were then trained and tested using the ‘balanced’ data. Using F1 scores as a metric, we observed the performances of each algorithm across the 30 subsampled datasets. We compared the F1 scores of our synthetically balanced datasets against those of the original datasets, and implemented hierarchical clustering to group the algorithms based on their overall stability. Algorithms that are not determined to be part of the large cluster of algorithms with small changes in F1 score are considered unstable.

### Under-represented cell type detection analysis

To evaluate the ability of each algorithm to identify specific cell types that represent a small portion of a dataset, we selected all cell types containing fewer than 1000 and comprising less than 30% of a dataset, and cell types comprising less than 5% of a dataset. We then assessed how the algorithms perform at detecting under-represented cell populations.

**Key Points**

- Cell-based fully supervised methods generally achieve higher performance and are faster compared to cluster-based methods.
- Cluster-based methods label non-malignant cell types more successfully than malignant cells, likely because of a lack of gene signatures for relevant malignant cell subpopulations.
- Examining the stability of labelling algorithm and their ability in consistently identifying all cell types with varying sizes are crucial for selecting right labelling algorithms.
- SVM and scPred and singleR outperform other algorithms in detecting under-represented cell types.
- SVM and scPred show the best overall performances with cancer-specific data.

## Supplementary Material

Christensen-Supplemental-Data-Final_bbac561

## Data Availability

The data underling this article have been made available in a consistent format in our TMExplorer single-cell RNA-seq database and search tool [34]. The accession numbers (i.e. Gene Expression Omnibus (GEO), ArrayExpress (AE) or Genome Sequence Archive (GSA)) of all datasets are provided in the Methods and the Supplementary Table S3. The source codes of the analyses performed in this study are available in GitHub at https://github.com/shooshtarilab/scRNAseq-Automated-Cell-Type-Labelling.

## Notes

**Erik Christensen** is a master’s student at the Department of Computer Science, University of Western Ontario, under the supervision of Dr. Parisa Shooshtari. His research is focused on developing and applying computational methods for the analysis of cancer single-cell RNA-seq data.

**Dr Ping Luo** is a postdoctoral fellow at the Pugh Lab in Princess Margaret Cancer Centre, Toronto. His research focused on applying machine learning and network analysis models to study cancer multi-omics data.

**Dr Andrei Turinsky** is a bioinformatics manager at the Centre for Computational Medicine of the Hospital for Sick Children. His research interests include bioinformatics, data analysis and biomedical applications.

**Mia Husić** has a Masters in Cell and Systems Biology, University of Toronto, and has worked at the University of Toronto and The Hospital for Sick Children as a scientific writer.

**Alaina Mahalanabis** is a master’s student at the University of Waterloo, and a research assistant at the Center for Computational Medicine at SickKids Research Institute. Her research focuses on the analysis of single-cell RNA-seq data.

**Alaine Naidas** was formerly an undergraduate student at the Department of Pathology, University of Western Ontario, under the supervision of Dr Parisa Shooshtari. Her research focused on the identification of malignant cells in cancer single-cell RNA-seq data.

**Dr J. Javier Diaz-Mejia** is the Head of Data Science at Phenomic AI, leveraging bioinformatics and machine learning to study cancer stroma single cell biology. Formerly, he was a scientific associate at the University Health Network supervised by Dr Trevor Pugh developing tools to integrate and analyze cancer single cell data.

**Dr Michael Brudno** is a Professor of Computer Science at University of Toronto and Chief Data Scientist at University Health Network whose work investigates the capture of structured phenotypic data from clinical encounters and analysis of omics data.

**Dr Trever Pugh**, PhD, FACMG is a cancer genomics researcher, board-certified molecular geneticist and holder of the Canada Research Chair in Translational Genomics. As a Director of the Joint Genomics Program of the University Health Network and Ontario Institute for Cancer Research, he oversees a comprehensive platform to deliver basic, translational and clinical genomics services. He is also appointed as an associate professor in the University of Toronto Department of Medical Biophysics, senior scientist at the Princess Margaret Cancer Centre and senior investigator at the Ontario Institute for Cancer Research. His research lab is focused on understanding clinical implications of clonal shifts in cancer and non-cancerous cell populations during treatment, most recently using cell-free DNA, immune repertoire and single cell RNA-seq sequencing.

**Dr Arun Ramani** is the engineering manager and the Bioinformatics Lead at Invitae. He previously was the scientific director and the Bioinformatics Lead at the Center for Computational Medicine at the Hospital for SickKids Children. He utilizes various high-throughput omics data (exomes, genomes, transcriptomes, methylomes) to analyze, annotate, interpret, prioritize and ultimately improve diagnosis.

**Dr Parisa Shooshtari** is an assistant professor of Bioinformatics at the Departments of Pathology and Lab Medicine and Computer Science at the University of Western Ontario. She is also an investigator at the Ontario Institute for Cancer Research and an associate scientist at Lawson Health Research Institute. She leads initiatives on the computational, statistical and machine learning method development with a main focus on single-cell sequencing and multi-omics data integration.
