# Supplementary material for: Evaluation of single-cell RNAseq labelling algorithms using cancer datasets
Source: Brief Bioinform. 2022 Dec 30;24(1):bbac561. doi: 10.1093/bib/bbac561 (PMC9851326; doi:10.1093/bib/bbac561)
Supplement: Christensen-Supplemental-Data-Final_bbac561 [file christensen-supplemental-data-final_bbac561.zip › Christensen-Supplemental-Data-Final_bbac561.docx]

**Supplemental Figures and Tables**

Table of Contents

[Supplemental Figure 1: Heatmaps of multiple performance measures. 2](#_Toc110537276)

[Supplemental Figure 2: Pipeline for evaluating the influence of number of cells, genes and cell types on running time. 3](#_Toc110537277)

[Supplemental Figure 3: Heatmaps of per cell type F1 scores for each dataset. 7](#_Toc110537278)

[Supplemental Figure 4: Scatterplots showing the relationship between proportion of cells in a cell type and F1 score on that cell type. 9](#_Toc110537279)

[Supplemental Figure 5: Boxplots showing the number of cells and proportion of cells in each class. 10](#_Toc110537280)

[Supplemental Figure 6: The change in F1 score for various cell types when subsampled to different sizes. 12](#_Toc110537281)

[Supplemental Figure 7: A heatmap showing the F1 scores of all algorithms on the most under-represented cell types in all datasets. 13](#_Toc110537282)

[Supplemental Figure 8: Correlation between various metrics tested for assessing performance of cell type labelling methods. 14](#_Toc110537283)

[Supplemental Figure 9: Heatmaps of per cell category F1 scores for each dataset. 17](#_Toc110537284)

[Supplemental Table 1: Relationship between the 8 datasets and the labelling algorithms (Part 1). 18](#_Toc110537285)

[Supplemental Table 2: Relationship between the 8 datasets and the labelling algorithms (Part 2). 20](#_Toc110537286)

[Supplemental Table 3: Summary of cancer datasets used. 21](#_Toc110537287)

A B


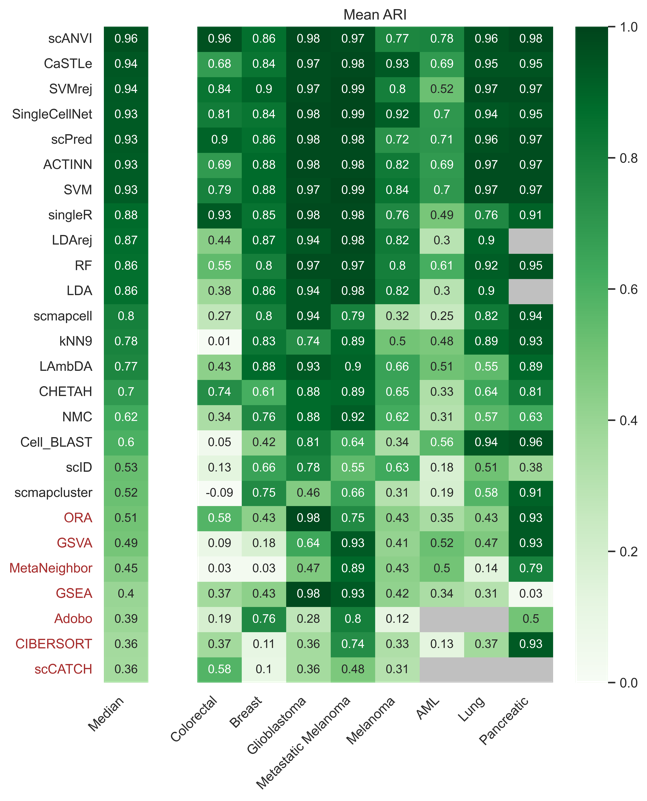

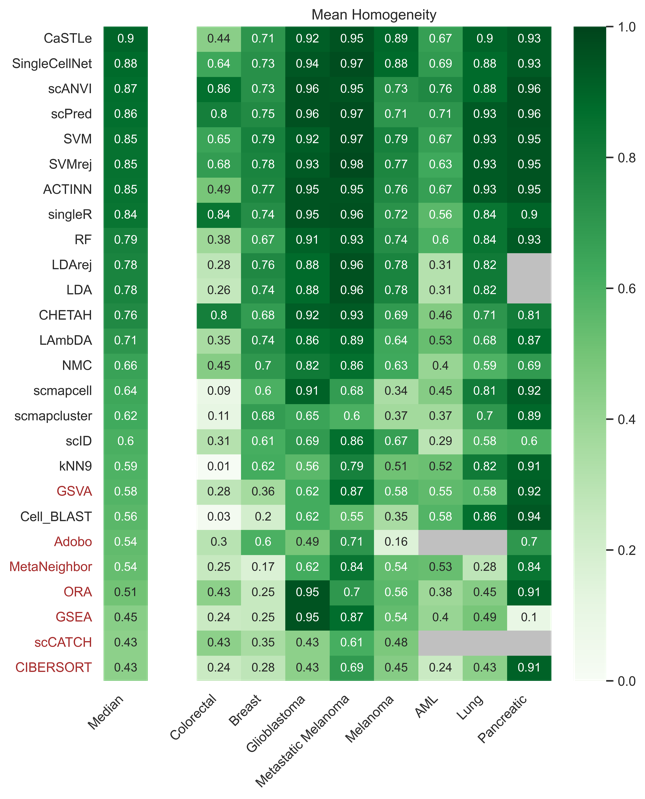


C D

**
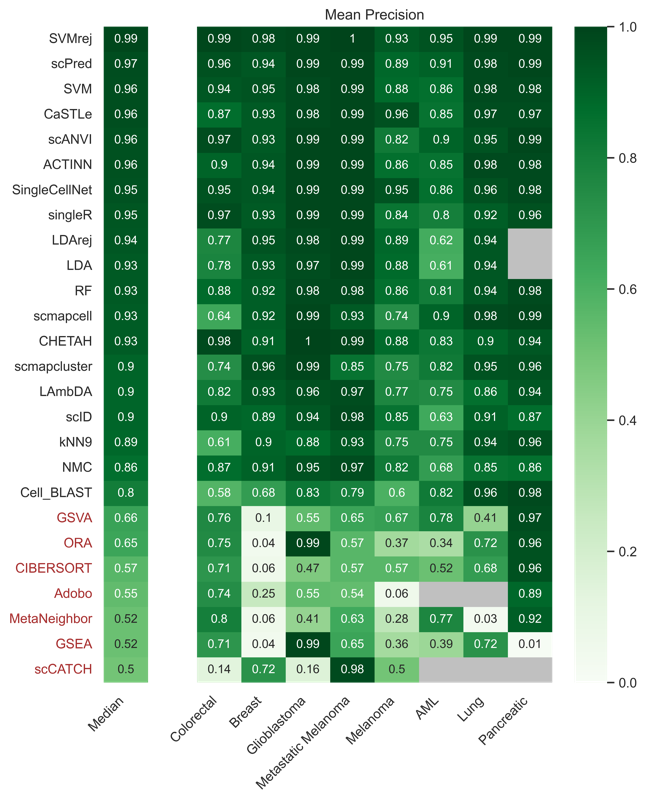

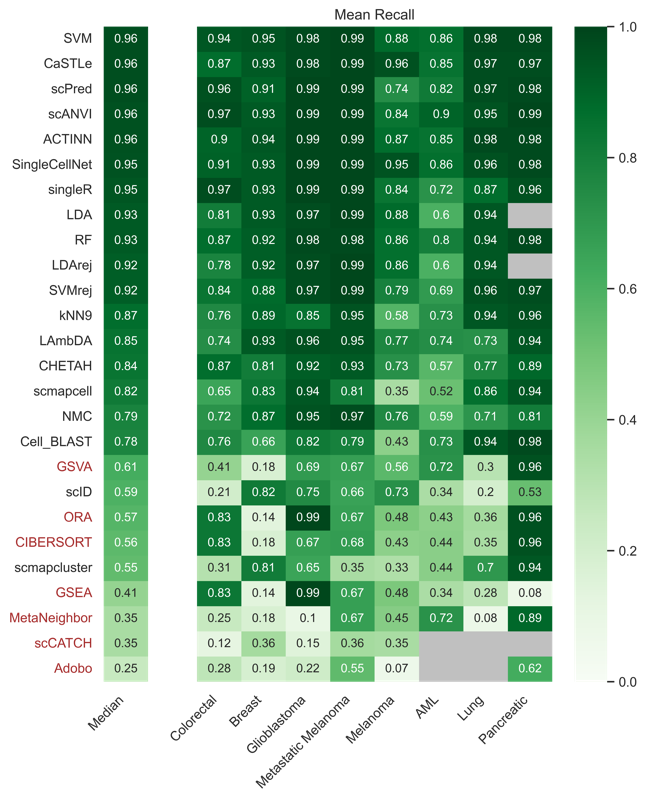
**

# **Supplemental Figure 1: Heatmaps of multiple performance measures.**

Heatmaps of mean (A) adjusted rand index (ARI), (B) homogeneity score, (C) precision, and recall (D) are shown. All measures were found to correlate with F1 Score. In each figure, the x-axis shows various datasets and the y-axis shows the different algorithms. Algorithms in black are cell-based, those in red are cluster-based.


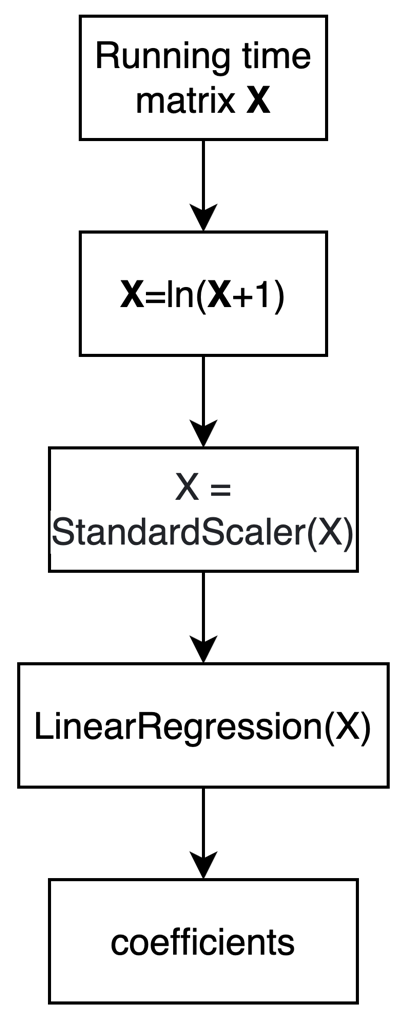


# **Supplemental Figure 2: Pipeline for evaluating the influence of number of cells, genes and cell types on running time.**

As our datasets vary in terms of numbers of cells, genes and cell types, we have evaluated the influence of these properties on running. Specifically, for each algorithm, a linear regression model was trained using the numbers of cells, genes and cell types as features, and the corresponding running time (ln(1+second)) as targets. Then, the coefficients of the learned models were calculated.

A

**
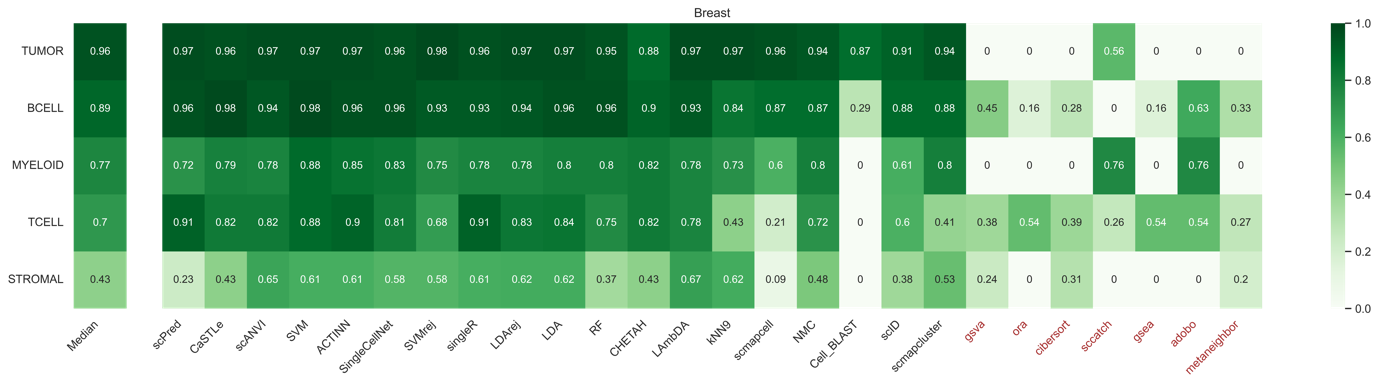
**

B


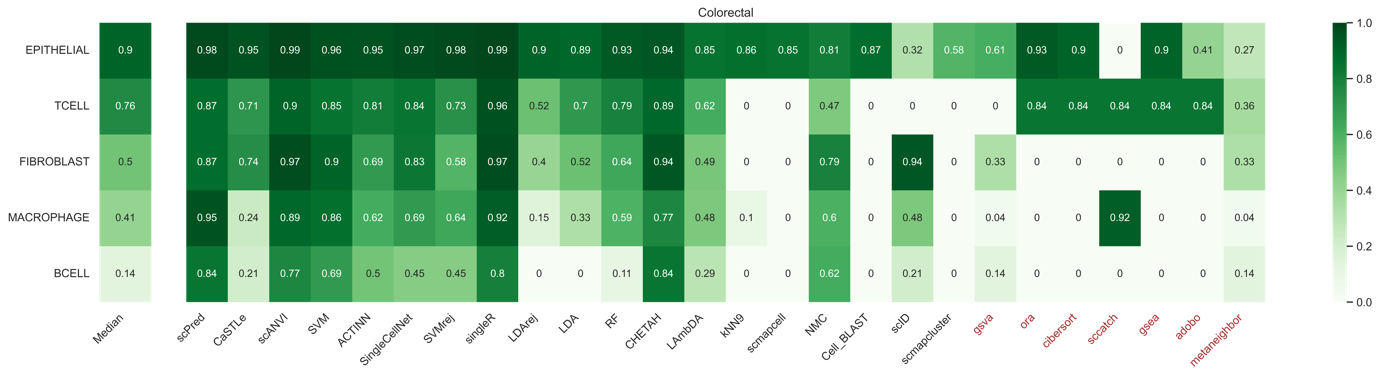


C


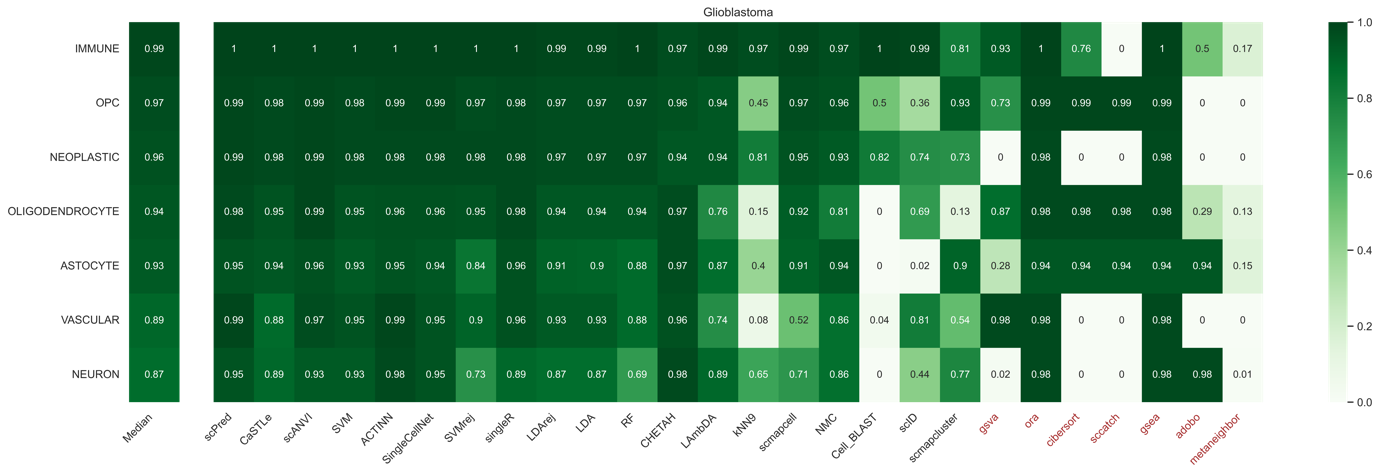


D


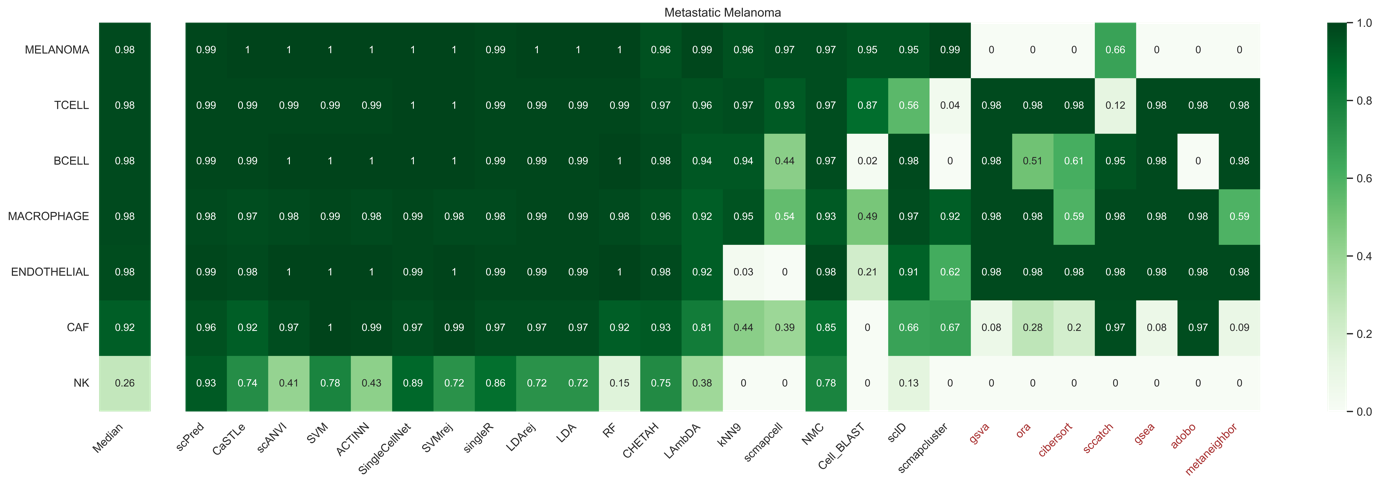


E


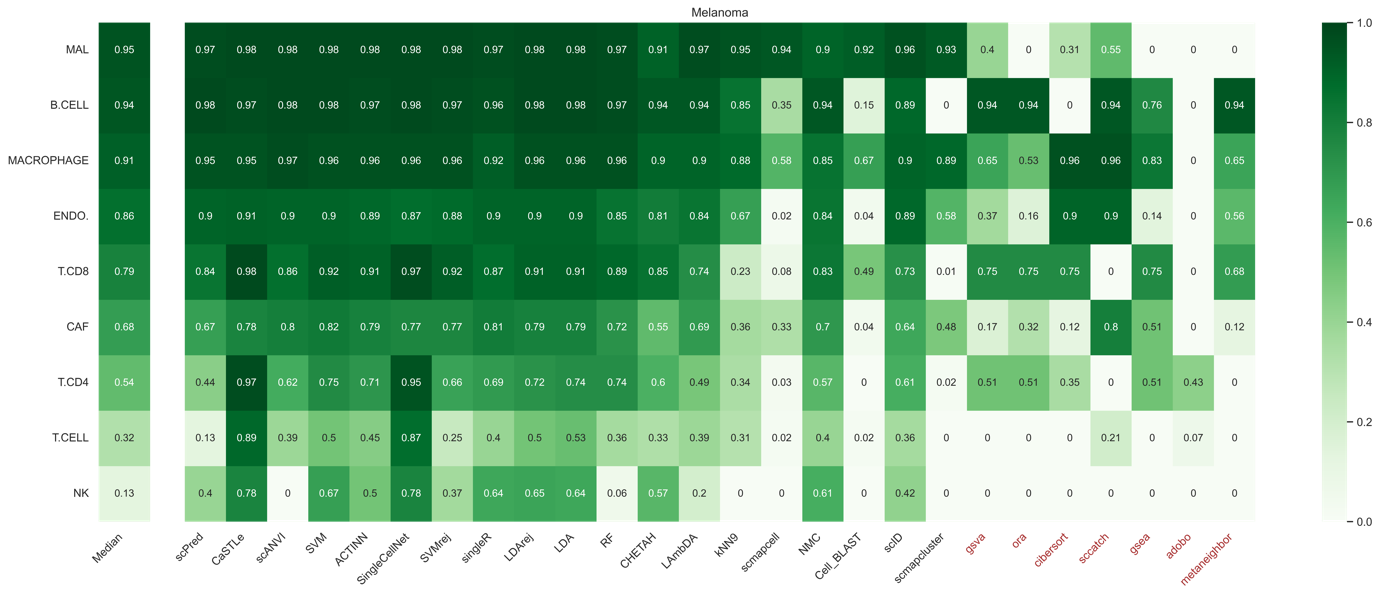


F


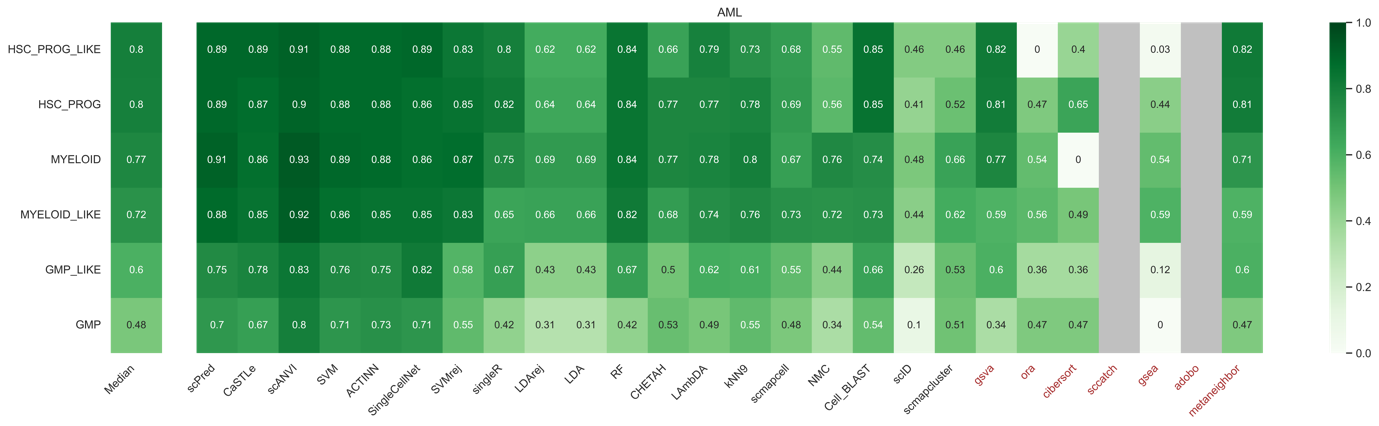


G


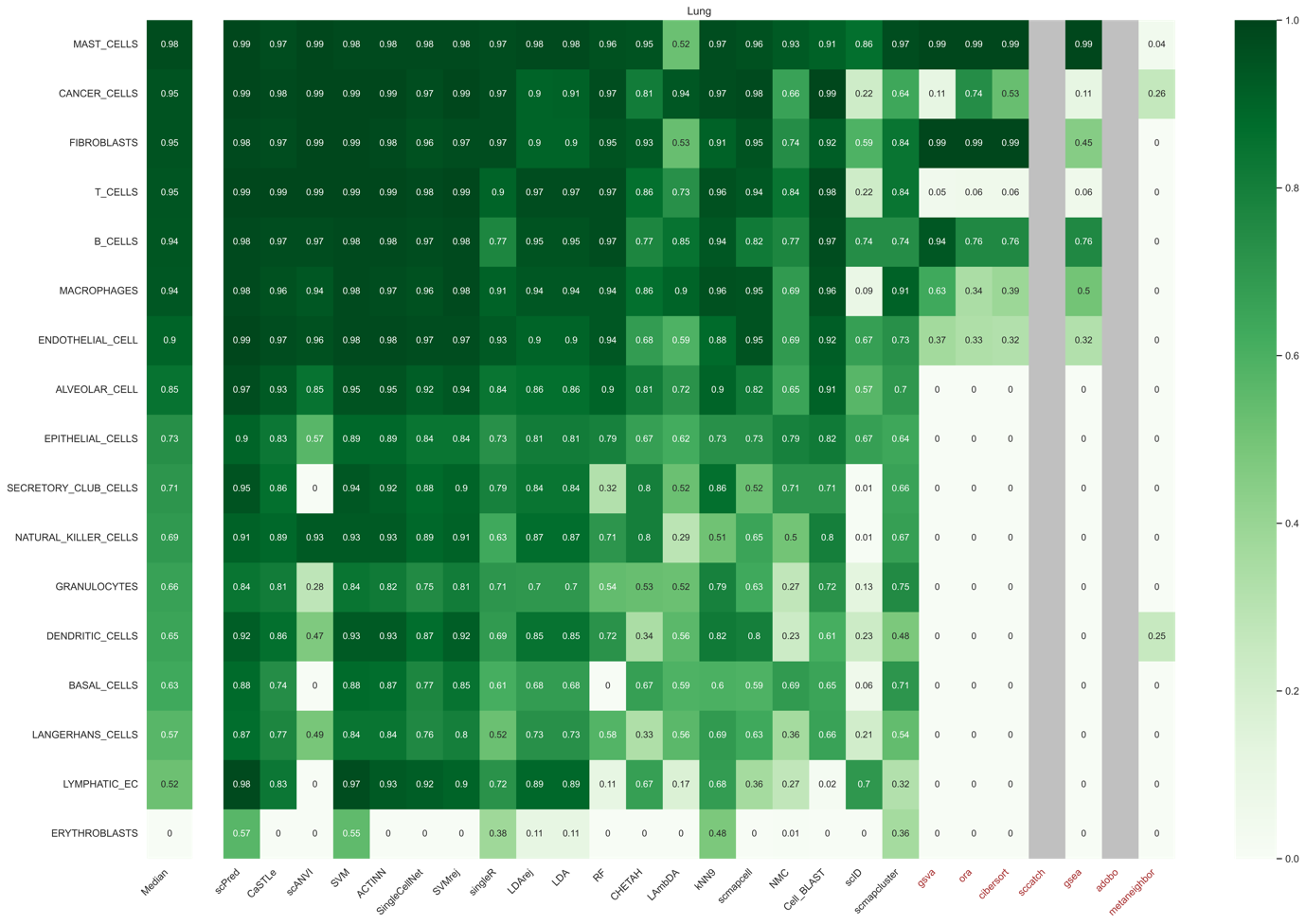


H

**
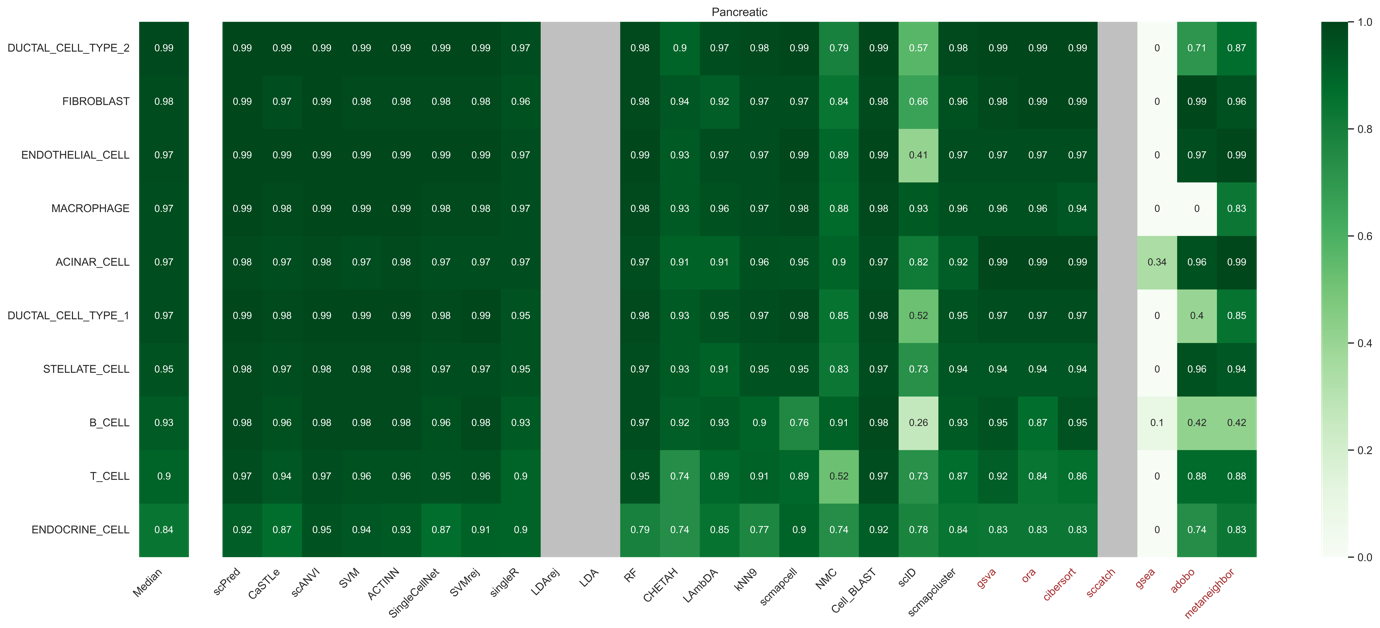
**

# **Supplemental Figure 3: Heatmaps of per cell type F1 scores for each dataset.**

F1 score heatmaps are shown for (A) Breast cancer, (B) Colorectal cancer, (C) Glioblastoma, (D) Metastatic Melanoma, (E) Melanoma, (F) AML, (G) Lung cancer, and (H) Pancreatic cancer datasets. In each figure, the x-axis shows different algorithms, and the y-axis shows different cell types (e.g. T-cells, B-cells, endothelial cells, etc.).

A
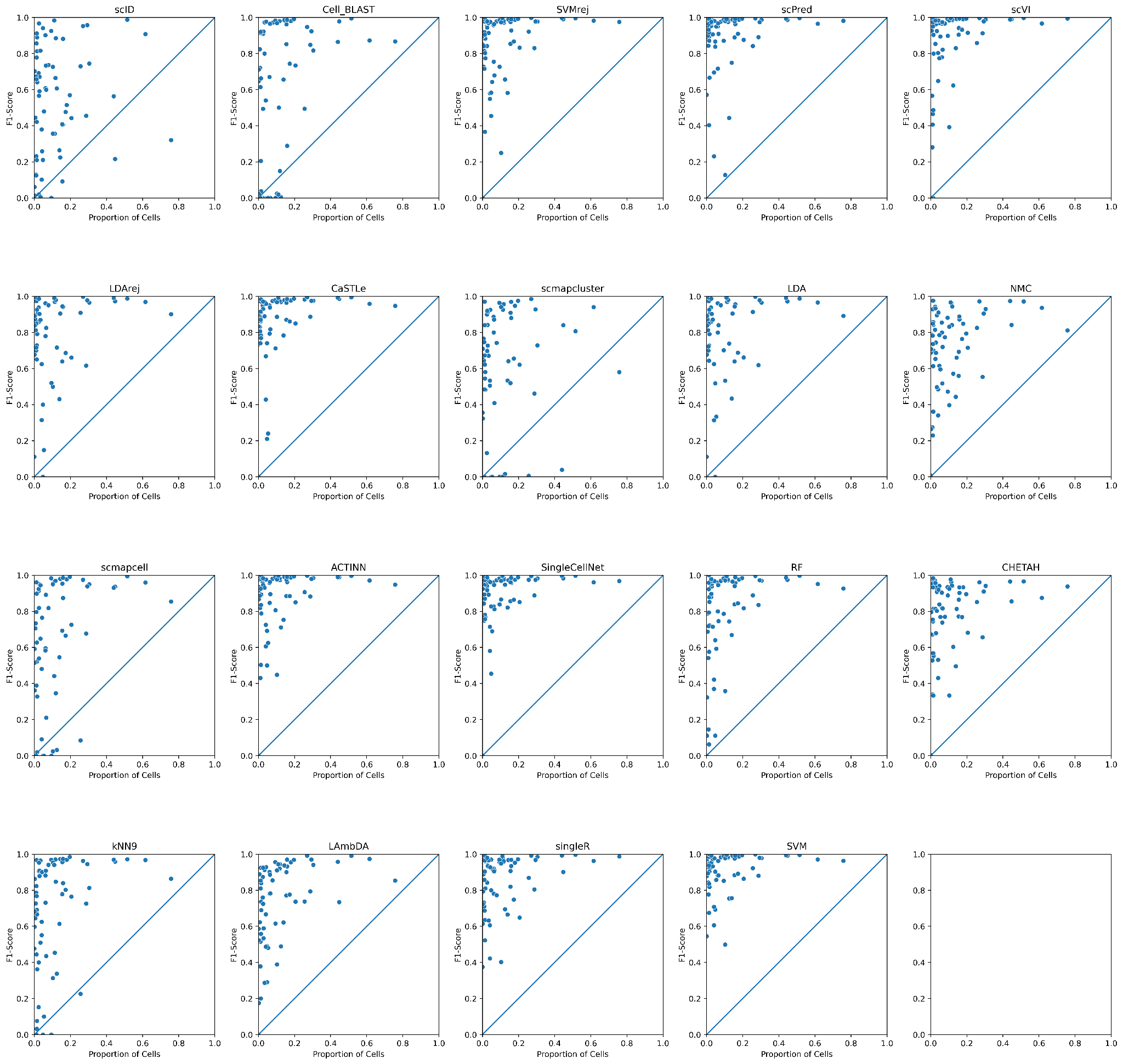


B
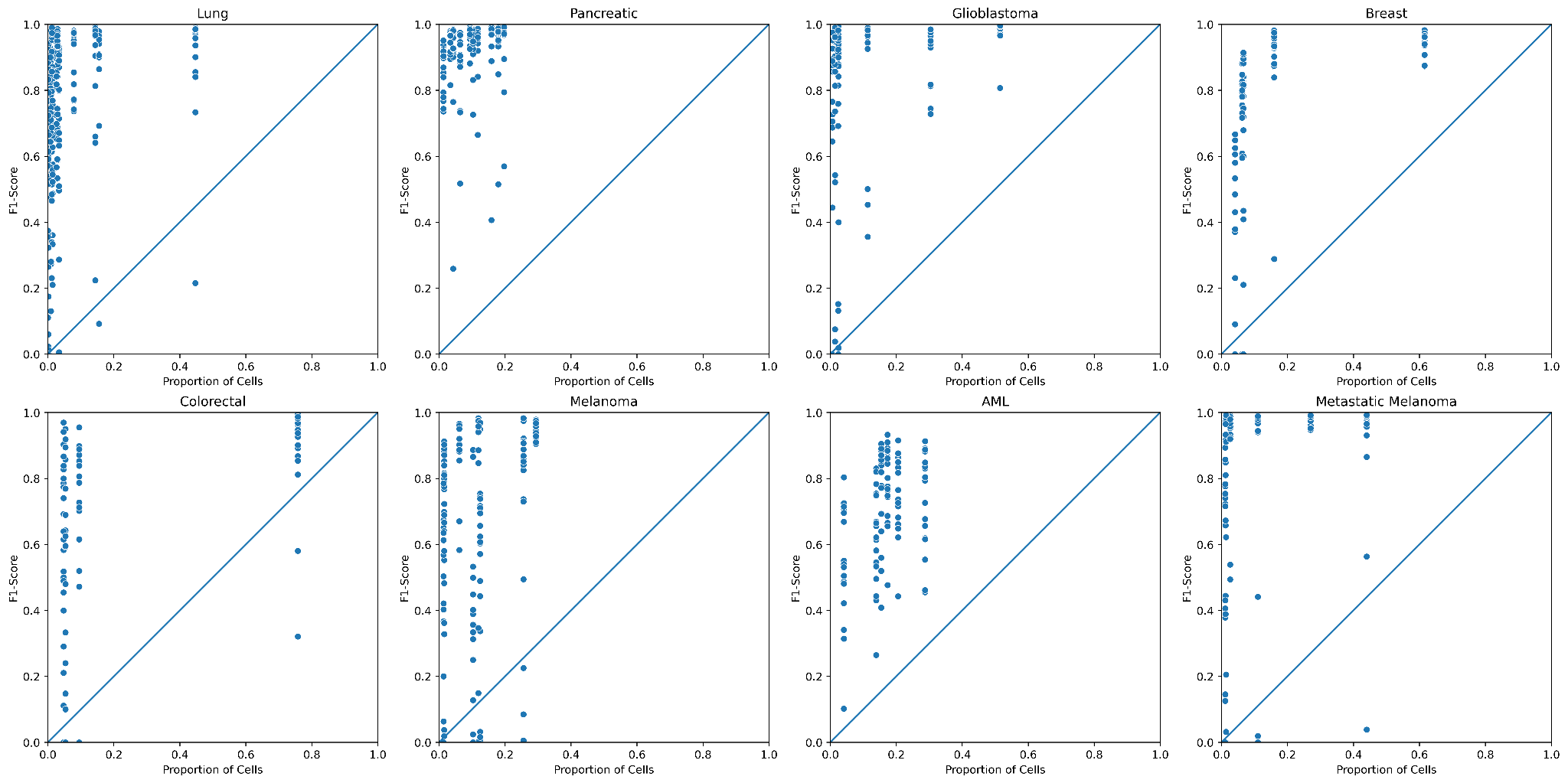


# **Supplemental Figure 4: Scatterplots showing the relationship between proportion of cells in a cell type and F1 score on that cell type.**

(A) all datasets across algorithms and (B) all algorithms across datasets.

**A B**


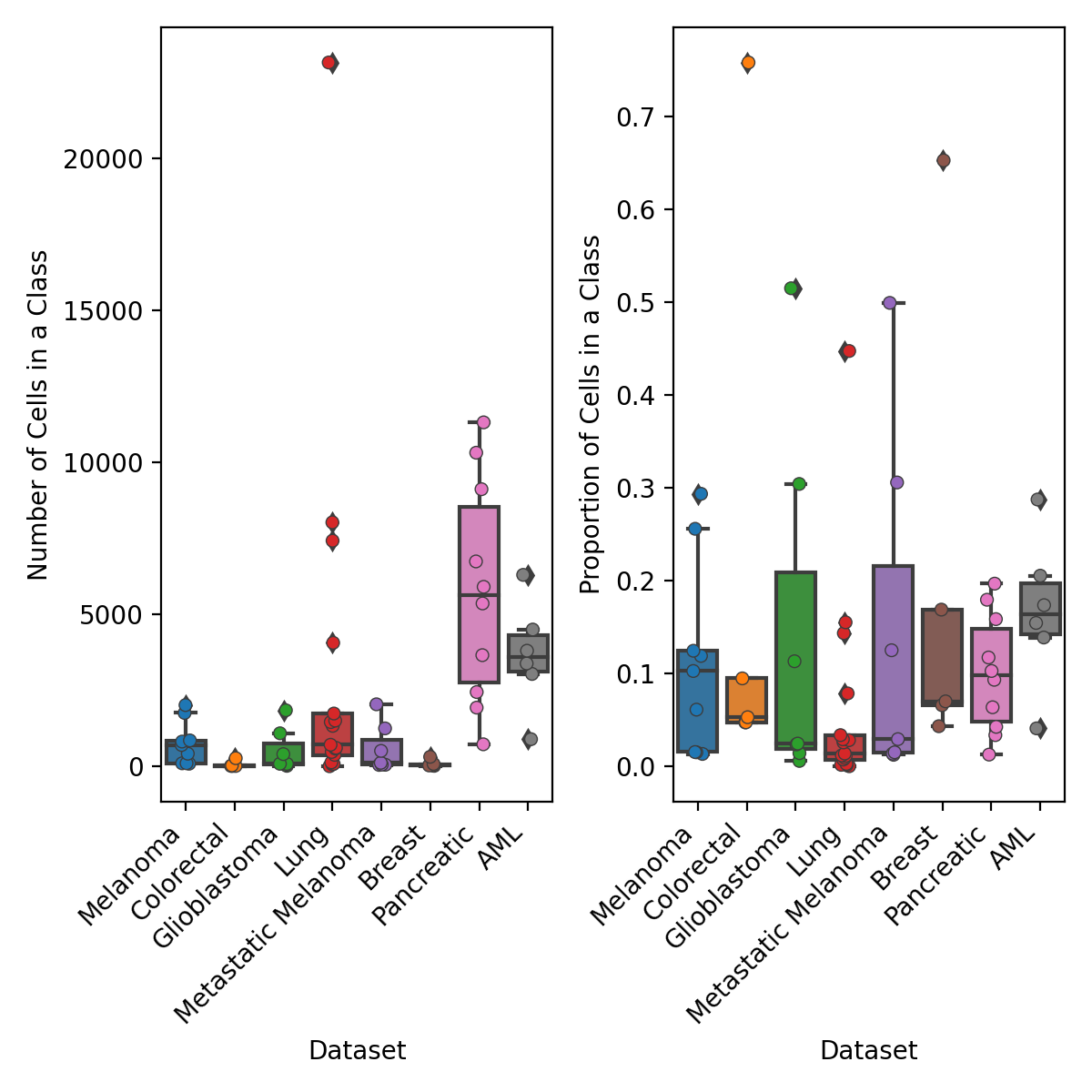


# **Supplemental Figure 5: Boxplots showing the number of cells and proportion of cells in each class.**

This figure demonstrates the imbalance in frequency we noticed between different cell types in some datasets. The Lung dataset, for instance, has one cell type comprising roughly 23,000 cells, about 45% of the dataset, while most other cell types are less than 5% of the dataset. We reasoned that the choice of algorithm affects performance on an imbalanced dataset more than the dataset itself since among multiple datasets where the largest class represents >40% of the data and smaller classes represent <5%, the only algorithms that were consistently affected were Cell_BLAST and scID.

**
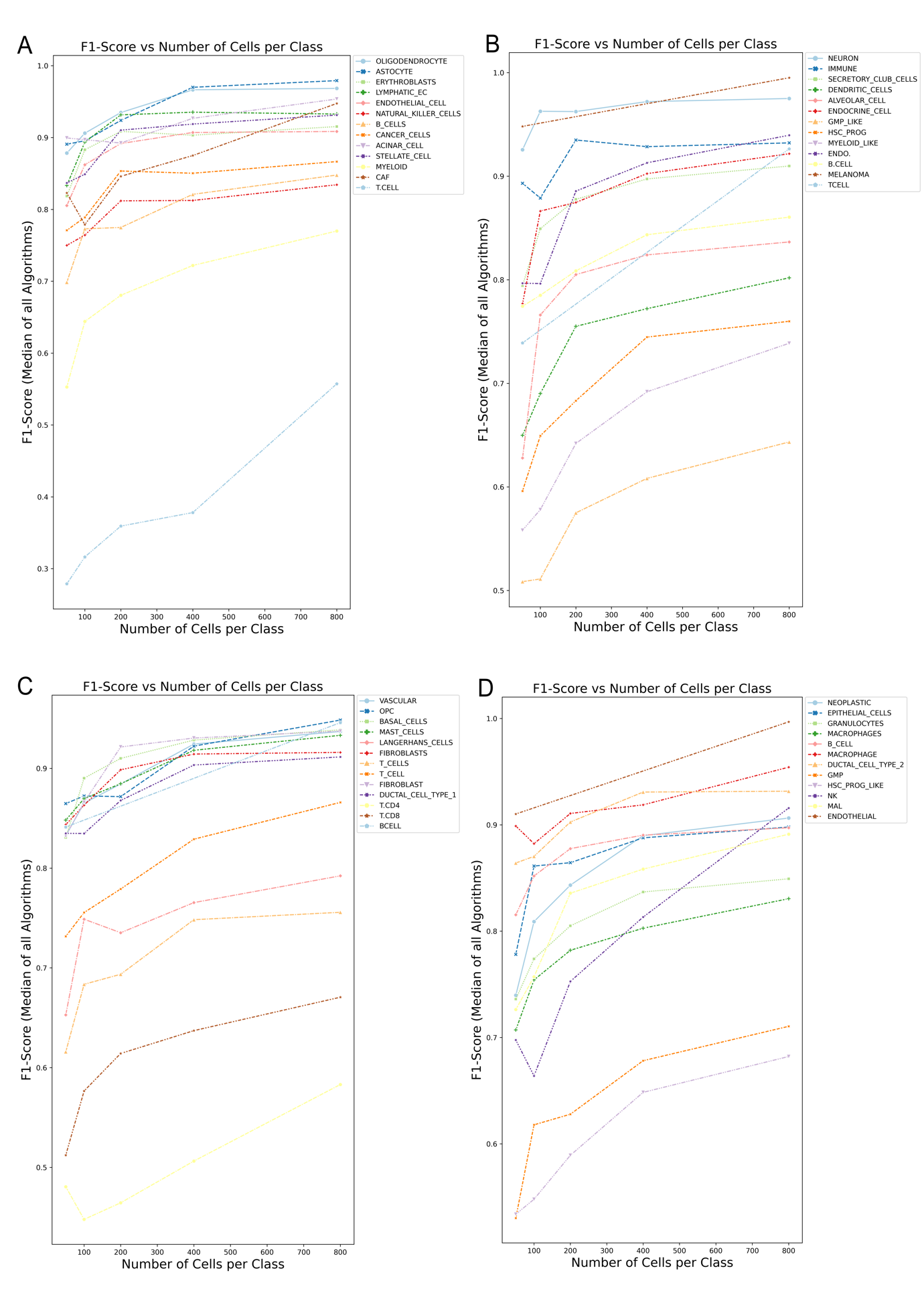
**

# **Supplemental Figure 6: The change in F1 score for various cell types when subsampled to different sizes.**

The x-axis shows the number of cells per class, and the y-axis shows the median F1 score across all algorithms. Each sub-figure (A, B, C, D) shows the result of 10-11 cell types.


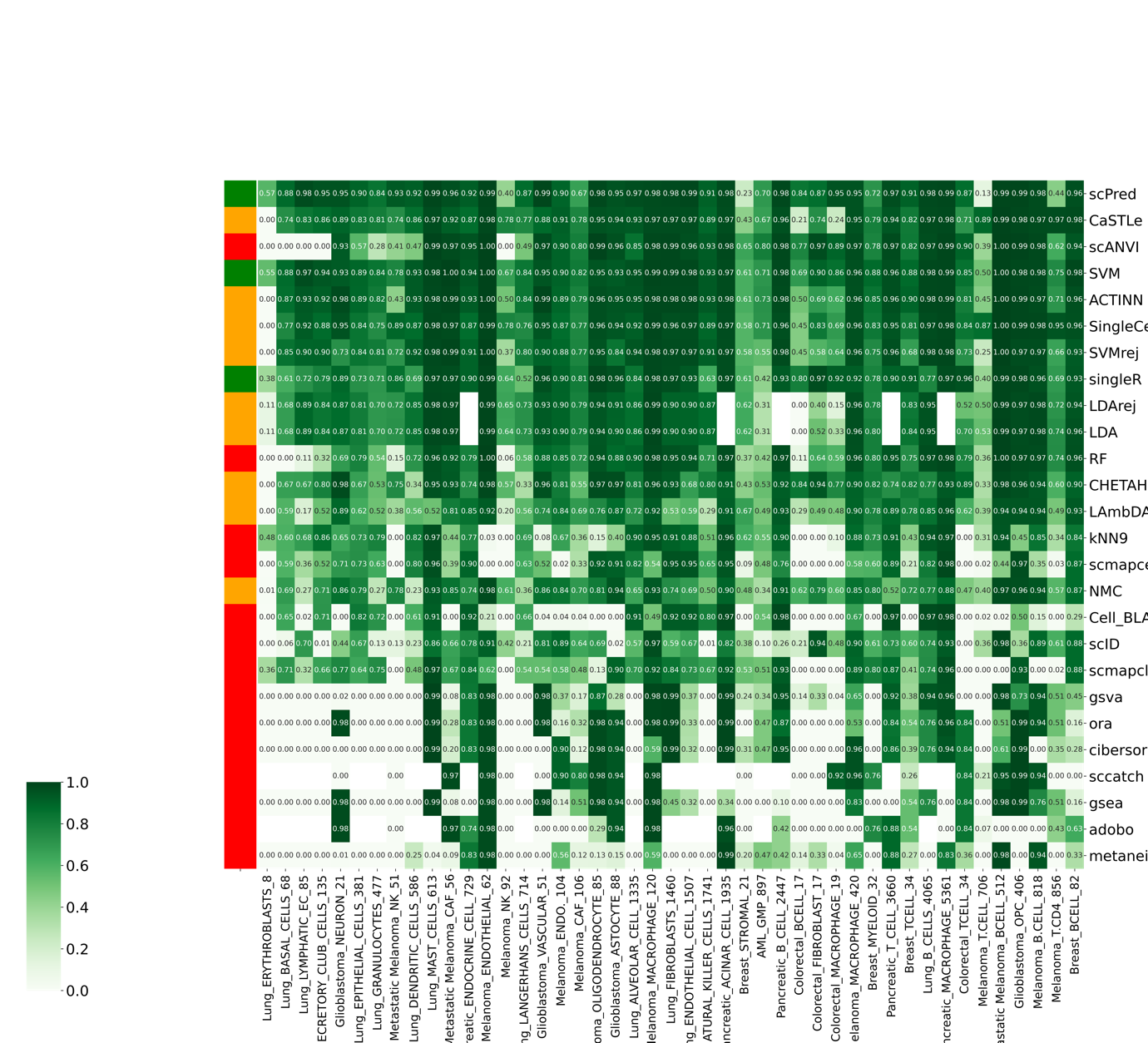


# **Supplemental Figure 7: A heatmap showing the F1 scores of all algorithms on the most under-represented cell types in all datasets.**

Under-represented cell types are those that are fewer than 1000 cells and less than 30% of the dataset, or those that are less than 5% of the entire dataset. Algorithms are assigned the colour green, orange, or red according to whether they identify underrepresented cells, fail to identify underrepresented cells, or only fail to identify underrepresented cells in extreme cases.


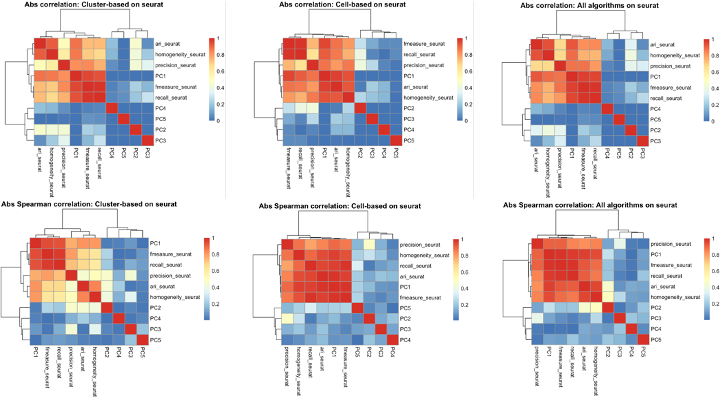


# **Supplemental Figure 8: Correlation between various metrics tested for assessing performance of cell type labelling methods.**

Each cell represents a correlation between two different metrics or principal components and the panels show that all metrics are correlated with F1 score, allowing us to use F1 score as our primary performance metric.

A


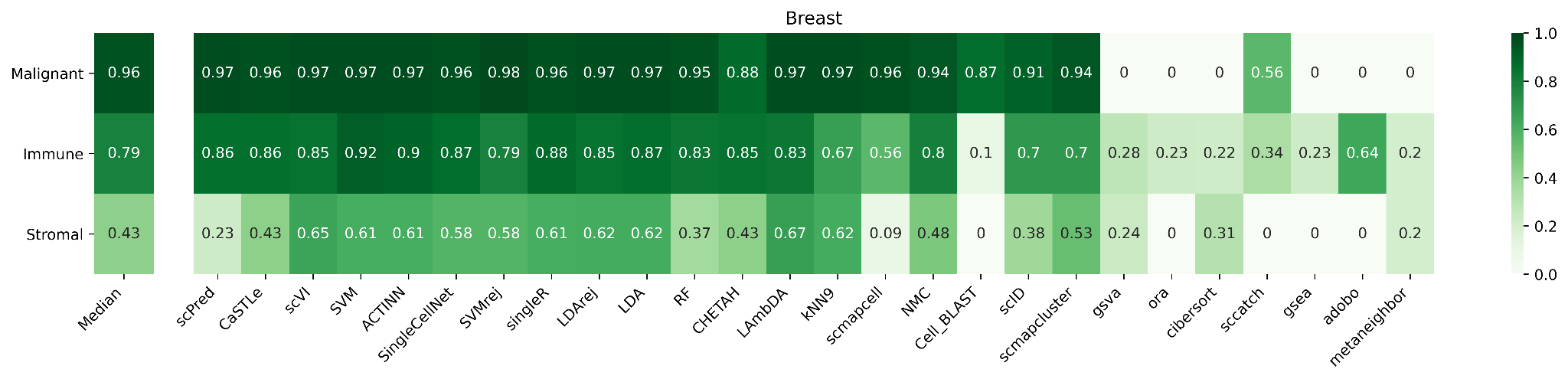


B


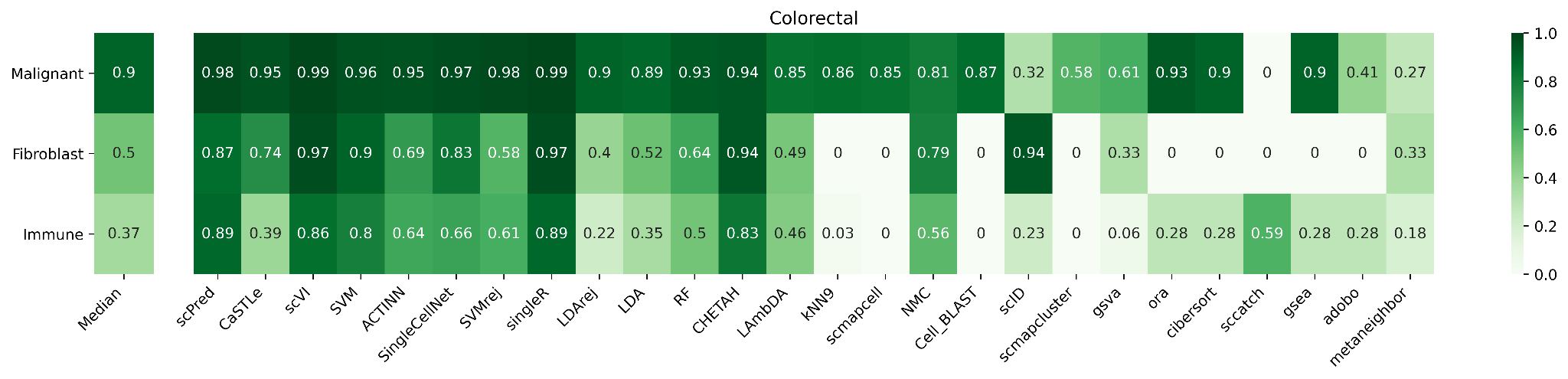


C


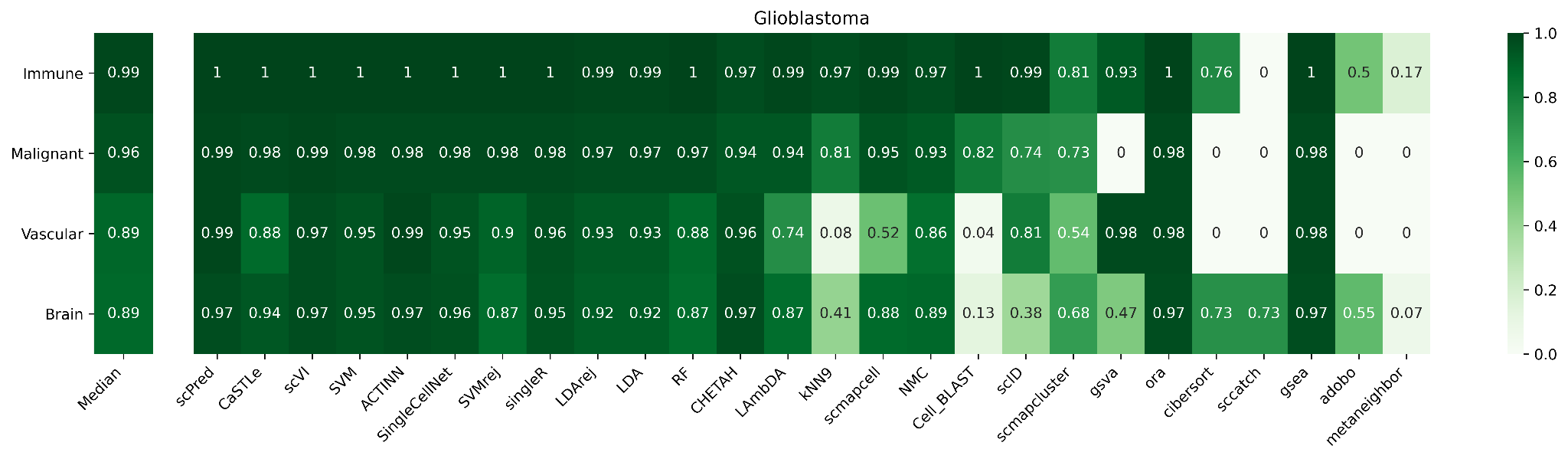


D


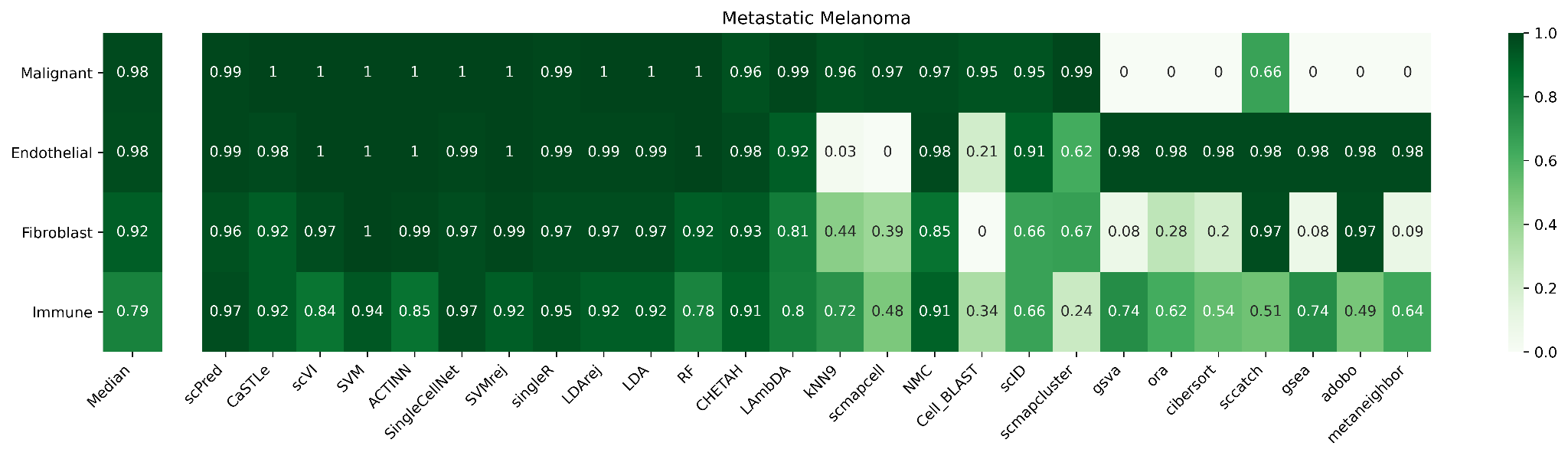


E


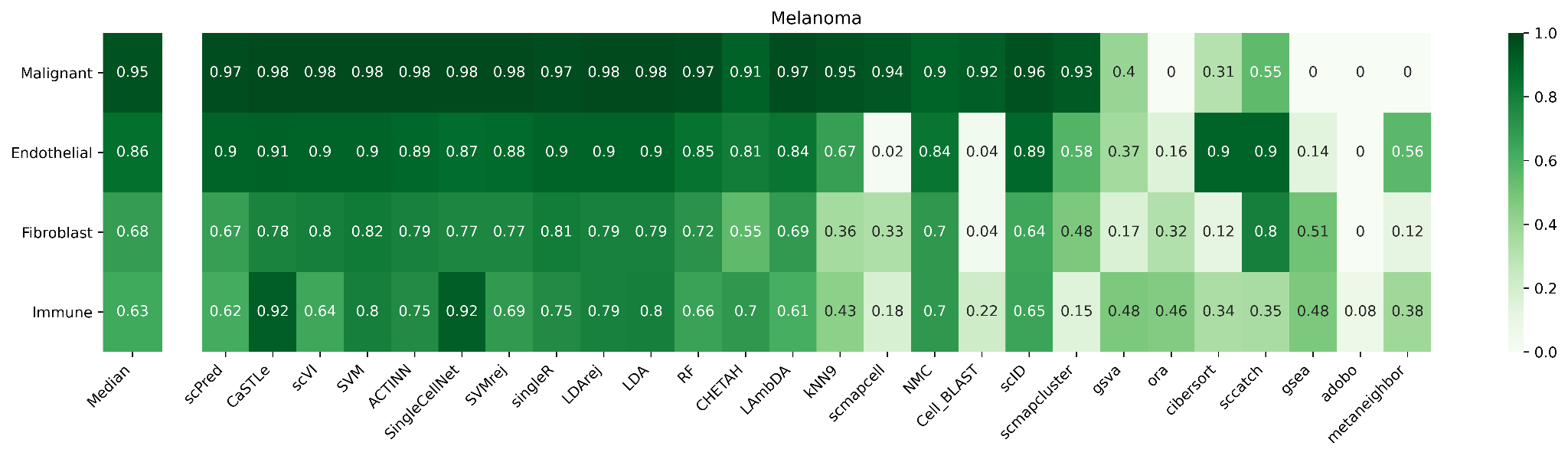


F


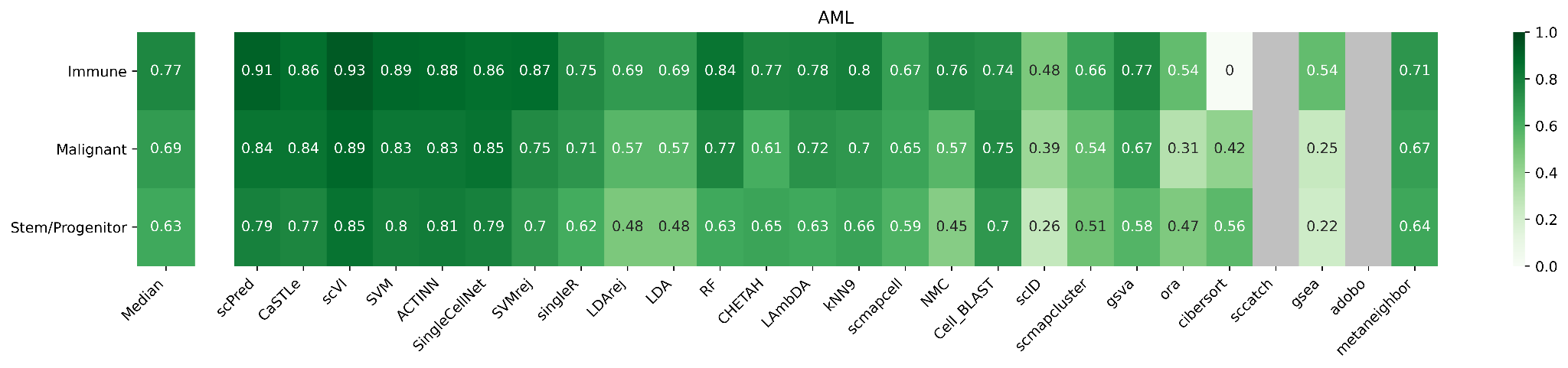


G


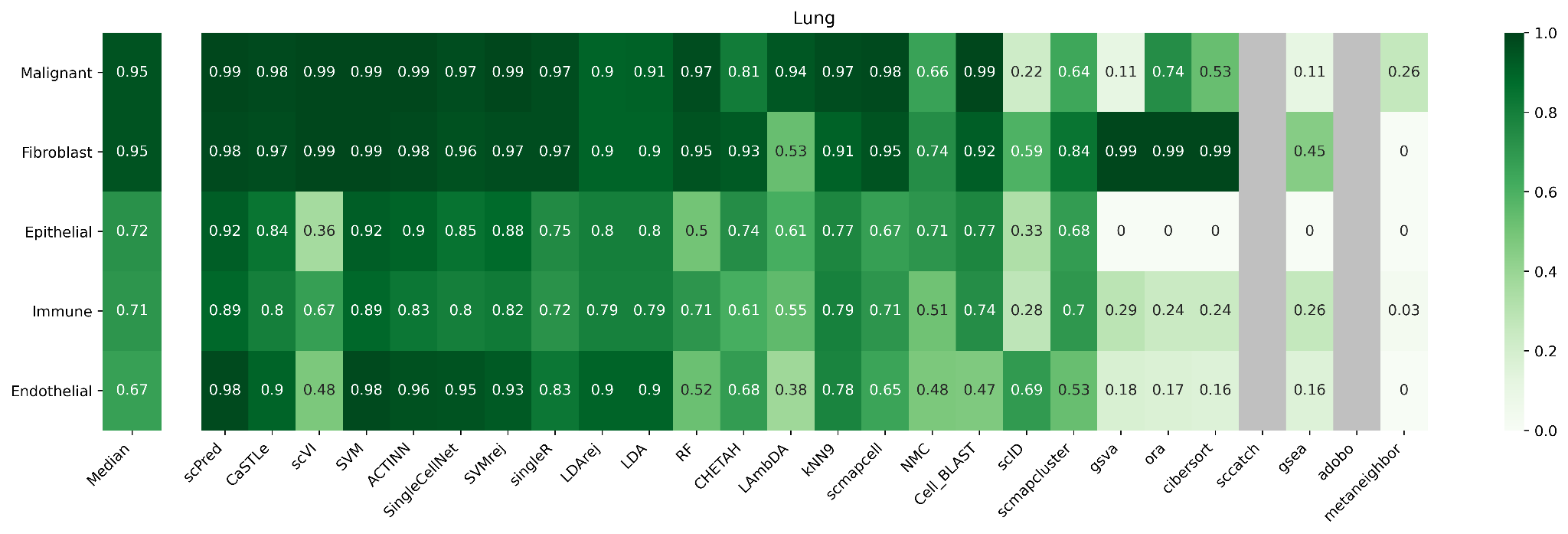


H


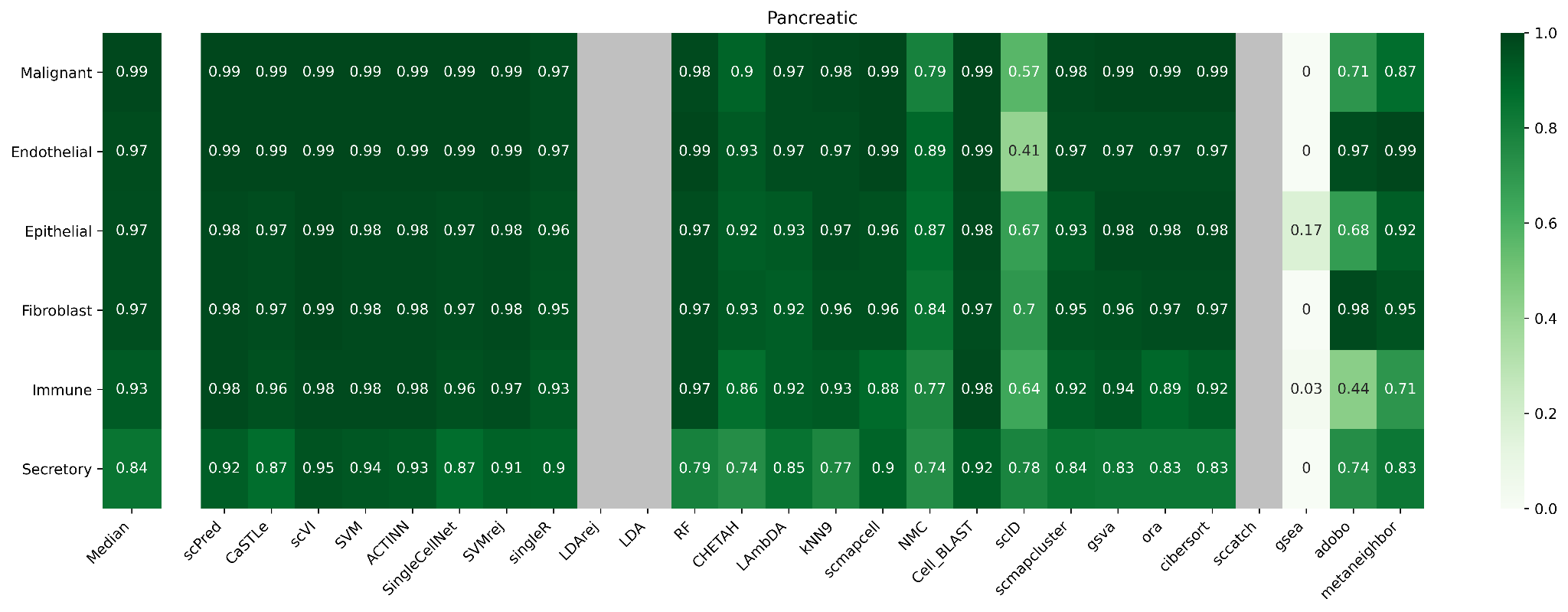


# **Supplemental Figure 9: Heatmaps of per cell category F1 scores for each dataset.**

Heatmaps of per cell type category F1 scores are shown for (A) Breast cancer, (B) Colorectal cancer, (C) Glioblastoma, (D) Metastatic Melanoma, (E) Melanoma, (F) AML, (G) Lung cancer, and (H) Pancreatic cancer datasets. In each figure, the x-axis shows different algorithms, and the y-axis shows different cell type categories (e.g. immune, epithelial, malignant, etc.).

# **Supplemental Table 1: Relationship between the 8 datasets and the labelling algorithms (Part 1).**

We examined whether the original studies that generated the 8 datasets had used any of the automated labelling algorithms to assign cell type labels to the cells.

| **Datasets used in the current study** | **Data analysis steps in the original publication, where the dataset was generated** | **Did the original study use any of our benchmarked labelling algorithms for their cell type annotation?** |
| --- | --- | --- |
| Pancreatic | Principal component analysis (PCA) followed by a graph-based clustering was used to identify clusters of cells. Manual gene signature analysis was used to assign cell type labels to the clusters. | No |
| Lung | Principal component analysis (PCA) and graph-based clustering were used to identify clusters of cells. Manual gene signature analysis was used to assign cell type labels to clusters of cells. | No |
| AML | Data was analyzed by using T-distributed stochastic neighbor embedding (tSNE) and mutation-specific single-cell genotyping. Cells were annotated by using random forest (RF) machine learning classifier. | Yes. Random Forest (RF) |
| Melanoma | CNV inferencing (inferCNV) and manual gene signature analysis were used for annotating cells. The results were compared to bulk RNA-seq profiles of cancer and normal tissue. | No |
| Metastatic Melanoma | CNV inferencing (inferCNV) and density-based clustering after data reduction by tSNE (T-distributed stochastic neighbor embedding) were used. Clusters of cells were labelled by using the manual gene signature analysis. | No |
| Glioblastoma | T-distributed stochastic neighbor embedding (tSNE) was used to reduce dimensionality of the data. This was followed by applying K-means clustering to cluster cells. Manual gene signature analysis and CNV inferencing were used for annotating clusters of cells. | No |
| Breast cancer | CNV inferencing was followed by hierarchical clustering of cells. Manual gene signature analysis was used for annotating clusters of cells. | No |
| Colorectal cancer | Reference component analysis was used for clustering cells. Manual gene signature analysis was used for assigning cell type labels to clusters of cells. | No |

# **Supplemental Table 2: Relationship between the 8 datasets and the labelling algorithms (Part 2).**

We assessed whether any of the algorithms identified as the top performers by our study used any of the 8 datasets in their original study.

| **Top 11 Algorithms** | **Which of the 8 datasets were used in the evaluation of the algorithm in the original study?** |
| --- | --- |
| scANVI | None |
| scPred | Colorectal cancer |
| ACTINN | None |
| CaSTLe | None |
| SingleCelNet | None |
| SingleR | None |
| SVM, SVMrej, LDA, LDArej, RF | None |

# **Supplemental Table 3: Summary of cancer datasets used.**

Summary of the datasets used to evaluate scRNA-seq labelling algorithms. Cancer type, number of cells, genes and tumors, sequencing technology used, annotation and gene signature availability and dataset accession numbers are provided. GEO: Gene Expression Omnibus; AE: ArrayExpress; and GSA: Genome Sequence Archive.

| **Dataset Name** | **Cancer type** | **Cells** | **Genes** | **Tumors** | **Sequencing technology** | **Cell types available?** | **Gene signatures available?** | **Accession number** |
| --- | --- | --- | --- | --- | --- | --- | --- | --- |
| Breast (Chung et al. 2017) | Primary breast  cancer | 515 | 57,915 | 11 | Fluidigm C1 | Yes | Yes | GEO: GSE75688 |
| Colorectal (Li et al. 2017) | Colorectal cancer | 359 | 57,241 | 11 | Fluidigm C1 | Yes | Yes | GEO: GSE81861 |
| Glioblastoma (Darmanis et al. 2017) | Primary glioblastoma | 3,589 | 23,465 | 4 | SMART-seq2 | Yes | Yes | GEO: GSE84465 |
| Melanoma (Jerby-Arnon et al. 2018) | Melanoma | 6,879 | 23,686 | 33 | SMART-seq2 | Yes | Yes | GEO: GSE115978 |
| Metastatic melanoma (Tirosh et al. 2016) | Metastatic melanoma | 4,645 | 23,686 | 19 | SMART-seq2 | Yes | Yes | GEO: GSE72056 |
| Lung (Lambrechts et al. 2018) | Non-small cell lung carcinoma | 51,775 | 22,533 | 5 | 10x Genomics | Yes | Yes | AE: E-MTAB-6149, E-MTAB-6653 |
| Pancreatic (Peng et al. 2019) | Pancreatic ductal adenocarcinoma | 57,530 | 24,005 | 24 tumors  11 controls | 10x Genomics | Yes | Yes | GSA: CRA001160 |
| AML (van Galen et al. 2019) | Acute myeloid leukemia | 21,933 | 27,899 | 40 | Seq-Well | Yes | Partial | GEO: GSE116256 |
